# Effects of dabigatran versus warfarin on 2-year cognitive outcomes in old patients with atrial fibrillation: results from the GIRAF randomized clinical trial

**DOI:** 10.1186/s12916-022-02563-2

**Published:** 2022-10-26

**Authors:** Bruno Caramelli, Pai Ching Yu, Francisco A. M. Cardozo, Iuri R. Magalhães, Raphael R. Spera, Daniel K. Amado, Maria C. Escalante-Rojas, Danielle M. Gualandro, Daniela Calderaro, Caio A. M. Tavares, Flavio A. Borges-Junior, Adriana F. Pastana, Mariana G. Matheus, Sonia M. D. Brucki, Ana Carolina O. Rodrigues, Ricardo Nitrini, Paulo Caramelli

**Affiliations:** 1 Unidade de Medicina Interdisciplinar em Cardiologia, Instituto do Coração, Hospital das Clínicas HCFMUSP, Faculdade de Medicina, Universidade de Sao Paulo, Av. Dr. Enéas C de Aguiar 44, São Paulo, 05403-000 Brazil; 2 Grupo de Neurologia Cognitiva e do Comportamento, Hospital das Clinicas HCFMUSP, Faculdade de Medicina, Universidade de Sao Paulo, Sao Paulo, SP Brazil; 3 Instituto do Coração, Hospital das Clínicas HCFMUSP, Faculdade de Medicina, Universidade de Sao Paulo, Sao Paulo, SP Brazil; 4 Behavioral and Cognitive Neurology Research Group, Faculdade de Medicina Universidade Federal de Minas Gerais, Belo Horizonte, MG Brazil

**Keywords:** Atrial fibrillation, Dementia, Cognitive scores, Dabigatran, Warfarin, Elderly

## Abstract

**Background:**

Observational studies support a role for oral anticoagulation to reduce the risk of dementia in atrial fibrillation patients, but conclusive data are lacking. Since dabigatran offers a more stable anticoagulation, we hypothesized it would reduce cognitive decline when compared to warfarin in old patients with atrial fibrillation.

**Methods:**

The GIRAF trial was a 24-month, randomized, parallel-group, controlled, open-label, hypothesis generating trial. The trial was done in six centers including a geriatric care unit, secondary and tertiary care cardiology hospitals in São Paulo, Brazil. We included patients aged ≥ 70 years and CHA2DS2-VASc score > 1. The primary endpoint was the absolute difference in cognitive performance at 2 years. Patients were assigned 1:1 to take dabigatran (110 or 150 mg twice daily) or warfarin, controlled by INR and followed for 24 months. Patients were evaluated at baseline and at 2 years with a comprehensive and thorough cognitive evaluation protocol of tests for different cognitive domains including the Montreal Cognitive Assessment (MoCA), Mini-Mental State Exam (MMSE), a composite neuropsychological test battery (NTB), and computer-generated tests (CGNT).

**Results:**

Between 2014 and 2019, 5523 participants were screened and 200 were assigned to dabigatran (*N* = 99) or warfarin (*N* = 101) treatment. After adjustment for age, log of years of education, and raw baseline score, the difference between the mean change from baseline in the dabigatran group minus warfarin group was − 0.12 for MMSE (95% confidence interval [CI] − 0.88 to 0.63; *P* = 0.75), 0.05 (95% CI − 0.07 to 0.18; *P* = 0.40) for NTB, − 0.15 (95% CI − 0.30 to 0.01; *P* = 0.06) for CGNT, and − 0.96 (95% CI − 1.80 to 0.13; *P* = 0.02) for MoCA, with higher values suggesting less cognitive decline in the warfarin group.

**Conclusions:**

For elderly patients with atrial fibrillation, and without cognitive compromise at baseline that did not have stroke and were adequately treated with warfarin (TTR of 70%) or dabigatran for 2 years, there was no statistical difference at 5% significance level in any of the cognitive outcomes after adjusting for multiple comparisons.

**Trial registration:**

Cognitive Impairment Related to Atrial Fibrillation Prevention Trial (GIRAF), NCT01994265.

**Supplementary Information:**

The online version contains supplementary material available at 10.1186/s12916-022-02563-2.

## Background

Atrial fibrillation (AF) prevalence and incidence increase with age [1]. In addition, AF is associated with an increased risk of cognitive decline and dementia [2], independently of shared risk factors or overt stroke. Several mechanisms might explain a causative role for cognitive impairment among individuals with AF, such as silent brain infarctions, cerebral microbleeds, and hypoperfusion [3, 4]. Despite limited and conflicting evidence [5], large observational studies [6, 7] support a role for oral anticoagulation to reduce the risk of dementia in AF patients, for whom effective therapeutic agents to mitigate its burden on healthcare systems [8] are needed.

Long-term oral anticoagulation therapy is currently recommended for patients with AF and a moderate-to-high risk of stroke, with non-vitamin K oral anticoagulants preferred over vitamin K anticoagulants (VKA) [1], due to significant risk reductions of systemic embolism and hemorrhagic stroke [9]. Cognitive outcomes, however, were not assessed in the pivotal randomized clinical trials (RCTs) that support this recommendation [10], including dabigatran etexilate, that was shown to be non-inferior to warfarin for the prevention of stroke and systemic embolism in the RE-LY trial (The Randomized Evaluation of Long-Term Anticoagulation Therapy) [10]. Observational studies added further uncertainty regarding the best strategy to prevent cognitive decline in patients with AF, as comparisons between non–vitamin K oral anticoagulants and VKA yielded different results [7, 11–13]. Since dabigatran has the theoretical advantage of a more stable anticoagulation status as compared to warfarin, it could improve cognitive related outcomes in patients with AF and at-risk of cognitive decline. Therefore, we conducted the CoGnitive Impairment Related to Atrial Fibrillation (GIRAF) randomized hypothesis generating trial comparing the use of dabigatran with warfarin in older adults with nonvalvular AF. We hypothesized that dabigatran would reduce cognitive decline, assessed by extensive cognitive test, independently of stroke.

## Methods

### Study design

The GIRAF trial was a 24-month, randomized, parallel-group, controlled, open-label, hypothesis generating trial, to compare dabigatran with warfarin in patients with AF or atrial flutter that was conducted in Sao Paulo, Brazil. The study protocol was approved by the local ethics committee and complies with ethics principles from the Declaration of Helsinki and International Conference on Harmonization Good Clinical.

GIRAF trial is an investigator-initiated research, partially funded by Boehringer Ingelheim do Brasil, which also provided dabigatran. The sponsor had no role in study design, trial execution, data analysis, writing/reviewing the manuscript, or in the submission for publication. Clinical Trial Registration: NCT01994265 (URL: www.clinical.trials.gov)

### Patients

Patients who were being followed at six centers in Sao Paulo (including a geriatric care unit, secondary and tertiary care cardiology hospitals), were invited to participate in the trial, but all the study procedures, including the final screening process, randomization, clinical and neurologic follow-up, and endpoints assessment, were performed at one site (Instituto do Coracao, HCFMUSP, Sao Paulo). Eligible patients were 70 years or older, had a history of AF or atrial flutter documented by a conventional 12-lead electrocardiogram (ECG) or by an ECG strip with duration of 30 seconds or longer, and had a CHA_2_DS_2_-VASc score of 2 or higher. Key exclusion criteria were illiteracy or less than 4 years of education, severe valvular heart disease (defined as any of the following anatomically severe valvular heart disease, per echocardiogram with compatible physical findings and cardiac auscultation: aortic stenosis/regurgitation, mitral regurgitation/stenosis, pulmonary regurgitation/stenosis or tricuspid regurgitation/stenosis), diagnosis of dementia (based on clinical judgment by the neurologist and on MMSE scores below education-adjusted norms for the Brazilian population), previous stroke or transient ischemic attack, severe liver disease, chronic kidney disease grade KDIGO 4 or worse (estimated glomerular filtration rate < 30 ml/min/1.73 m^2^), and major contraindications to oral anticoagulation. Full details of inclusion and exclusion criteria are available in the supplementary appendix.

### Randomization

After eligible patients provided informed consent, they were randomized 1:1 via a randomization program using the Research Electronic Data Capture (REDCap) system, to receive either open label dabigatran 150 mg or 110 mg twice daily (110 mg dose for patients ≥ 80 years or with an eGFR between 30 and 50 mL/min/1.73m^2^) or warfarin once daily titrated to achieve an international normalized ratio (INR) of 2.0 to 3.0.

### Procedures

Up to 15 days after randomization, all patients went through baseline cognitive evaluation. For patients who were using an oral anticoagulant before randomization other than its group assignment, switching to dabigatran was performed according to current guidelines [14]. For switching from dabigatran (or other non-vitamin K oral anticoagulant), warfarin was started according to the creatinine clearance: if ≥ 50 mL/min, 3 days before discontinuing non-vitamin K oral anticoagulant C, and 2 days before discontinuing it if the creatinine clearance was between 30 and 50 mL/min.

A pre-specified, comprehensive, and thorough cognitive evaluation for different cognitive domains was performed at baseline and at 24 months, based on the recommendations of the National Institute of Neurological Disorders and Stroke-Canadian Stroke Network Vascular Cognitive Impairment Harmonization Standards [15]. The Mini-Mental State Examination (MMSE) and the Montreal Cognitive Assessment (MoCA) were administered as brief measures of global cognitive functioning. In addition, participants were submitted to a neuropsychological test battery (NTB), including the following tests: Trail-Making tests A and B, short form (15 items) of the Boston naming test (BNT), clock drawing test (CDT), digit symbol substitution test (DSST), phonemic verbal fluency test (FAS), semantic verbal fluency test (SVF; animals/minute), and the Figure Memory Test (including immediate, learning, and delayed recall). Participants also underwent computer-generated neuropsychological tests (CGNT), which evaluated simple reaction time and sustained, selective and divided visual attention, with measures of accuracy (i.e., percentage of correct responses) and reaction time (in milliseconds). A detailed explanation of the CGNT can be found elsewhere [16]. All the tests have been used previously in Brazilian Portuguese versions. Cognitive evaluations lasted approximately 90 min and were performed by two neurologists, blinded to group assignments, in separate visit days from clinical consultations. Details regarding the through cognitive evaluation are provided in the supplementary appendix.

In patients randomized to open-label warfarin, INR was measured weekly until the INR goal, then bi-weekly and monthly if the drug dosing was stable and the INR remained within target range (2.0 to 3.0). The time that the INR was within the therapeutic range during the trial was calculated with the use of the method of Rosendaal et al. [17]. Clinical consultations were performed every 3 months for both groups.

### Outcomes

Primary outcomes were changes in cognitive performance at 24 months from baseline, measured with MoCA, MMSE, NTB, and CGNT scores as each test analyzes specific cognitive domains. Importantly, despite analyzing different domains of cerebral performance, all tests analyze cognitive function, that represents the main outcome of our study. The NTB and CGNT (accuracy and reaction time measures) scores were calculated as composites *Z*-scores, by averaging individual tests’ *Z*-scores weighted according to the number of available tests per patient. Prior to calculation, all components (tests) were standardized to indicate a better performance with higher scores (e.g., by using the negative of reaction time for the CGNT components). The minimum necessary number of components for calculating a patient’s composite score was set to six for the NTB score and seven for the CGNT. The respective value of the composite score was considered missing if the minimum number components condition was not met. Exploratory outcomes, based on post hoc analyses, were changes in cognitive domain scores for executive functioning, attention, language, and memory at 24 months in comparison to baseline. The executive functioning domain included the CDT, trails A and B. The attention domain included DSST and all CGNT tests. The language domain included the BNT, FAS, and SVF tests. The memory domain included the Figure Memory Test. The minimum necessary number of components for calculating the composite score was two for the executive functioning, language, and memory domains, and seven for the attention domain; a missing value was assigned otherwise. For all tests, cognitive decline was defined as any decline in *Z*-scores over time. Additional methods that were performed for neuroimaging for the diagnosis of silent stroke and biomarker assessments are described in the supplementary appendix.

### Statistical analyses

On the basis of a post-hoc analysis of two randomized controlled trials [18] and on clinical practice expertise of the authors, we estimated a mean drop of 2 points in the MMSE score after 24 months with a standard deviation of 2 points. Assuming a 10% dropout rate and similar between group differences at 24 months for all primary outcomes, we calculated that a sample size of 200 patients would provide our study a 80% power to detect a 50% difference of change in cognitive scores (measured by any of the primary outcomes) in patients treated with dabigatran compared to warfarin. These estimate were later further supported by a study [19] that estimated a mean 0.2 drop in the NTB *Z* score with standard deviation of 0.5.

Primary analysis was conducted according to the modified intention-to-treat (mITT) population, including all patients who underwent both baseline and 24-month cognitive evaluations, censoring for patients who had stroke or other cerebrovascular events throughout the study. Additional sensitivity analyses were also performed to test the consistency of our findings: first using a per-protocol analysis on the mITT population (excluding patients that switched or stopped their oral anticoagulation during the 24-month period and including all randomized patients that underwent the first cognitive evaluation) and second using regression-based multiple imputation to estimate missing values for at 24-month evaluations. A linear regression was carried out for each primary outcome with treatment (D or W, 0/1 coded), age (years), education (log of years), and baseline raw score as covariates, with no interaction factors. After individual analyses of the relationship of covariates and dependent variables, we found only weak linear relationships. Additional analyses of the residuals of the linear regressions disclosed no major discrepancies to the standard assumptions. We report the results as least-square mean changes from baseline for each group (higher scores indicate better cognitive performance) and as the difference between-groups, at baseline and 24 months, with 95% confidence intervals. Cohen’s *d* standardized size effects are also reported based on the mean treatment difference between groups and residual standard deviation. The confidence intervals and *P*-values reported refer to a two-sided alpha of 0.05 with no correction for multiple hypothesis testing. To account for the increased risk of a type 1 error in the multiple comparisons of primary endpoints, adjusted *P*-values were also computed using Hommel’s method and reported in the “Results” section. All statistical analysis were performed using the R software, version 4.1.2 (R Foundation for Statistical Computing), and graphics were elaborated using GraphPad Prism version 9.3.0 for Windows, GraphPad Software, San Diego, California USA, www.graphpad.com.

## Results

**Fig. 1:**
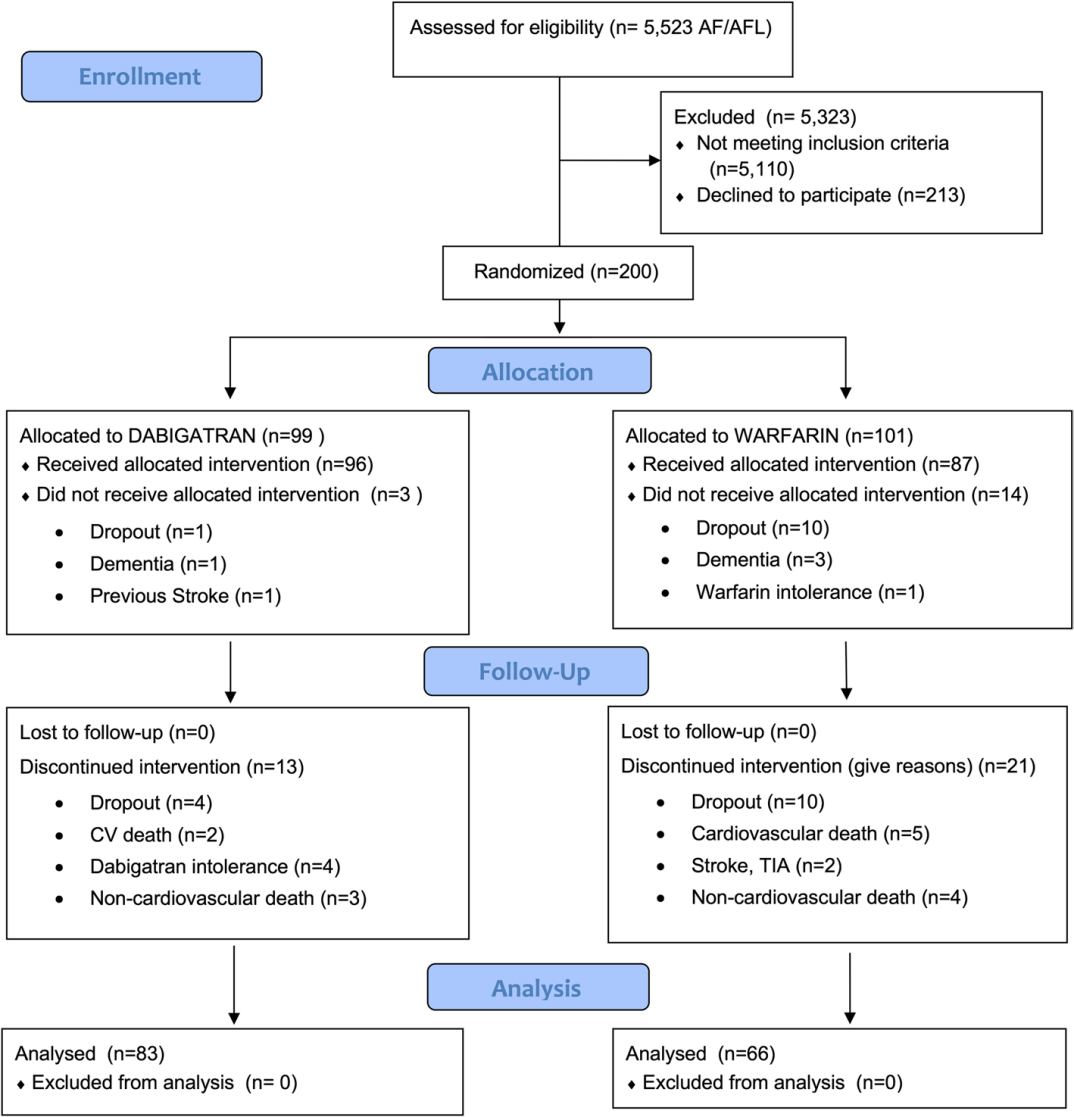
GIRAF study flowchart. The patient flowchart depicts those who completed the 2 years cognitive assessment, dropout, and developed intolerance to medication. AF, atrial fibrillation; AFL, atrial flutter; TIA, transient ischemic attack

Between November 7, 2014, and March 10, 2019, 5523 participants were screened and 200 patients already on previous anticoagulation for the prevention of stroke were randomly assigned to either dabigatran (*N* = 99) or warfarin (*N* = 101) treatment. The major reasons for ineligibility were prior valvular heart disease (28%) and prior TIA or stroke (18%). A full list of ineligibility criteria is shown in the supplementary appendix. The mITT analyses included 149 patients who completed the 2 years cognitive assessment (Fig. 1). There were no significant between-group differences at baseline regarding age, sex, years of education, MMSE, HAS-BLED and CHA_2_DS_2_-VASc scores. MoCA, NTB, and CGNT scores, however, were different between groups at baseline (Table 1, and see Additional file: Table S3-S11, Table S12 [20–23], and Figures S4-S10).

**Table 1:** Baseline characteristics of the patients included in the GIRAF trial. Data are depicted according to arm allocation for patients that completed the 2 years cognitive assessment (mITT population, no imputation). Numbers indicate median (IQR) for non-normal continuous variables, mean (standard deviation) for continuous variables, and number (percentage) for dichotomous variables. Normality was assessed by a Shapiro-Wilks test at 5% significance

Characteristics	Dabigatran (*n* = 83)	Warfarin (*n* = 66)
Age (years)	74 (71 to 77)	76 (72 to 77)
Sex (male)	51 (61.4%)	39 (59.1%)
Education (years)	7 (4 to 12)	4 (4 to 9)
CHA2DS2-VASc median (IQR)	4 (3 to 4)	4 (3 to 5)
HAS - BLED median (IQR)	1 (1 to 1.25)	1 (1 to 1)
MMSE score	27 (26 to 29)	27 (26 to 29)
MoCA score	23 (19 to 25)	22 (21 to 25)
NTB score	0.08 (0.60)	− 0.12 (0.58)
CGNT score (composite score)	0.24 (0.02 to 0.47)	− 0.04 (− 0.27 to 0.31)
Hypertension	72 (86.7%)	60 (90.9%)
Diabetes mellitus	30 (36.1%)	27 (40.9%)
Dyslipidemia	48 (57.8%)	37 (56.1%)
Tobacco (smoker + ex-smoker)	43 (51.8%)	30 (45.5%)
Coronary artery disease	18 (21.7%)	16 (24.2%)
Chronic heart failure	33 (39.8%)	32 (48.5%)
Hypothyroidism	25 (30.1%)	13 (19.7%)
Previous medications		
Acetylsalicylic acid	10 (12%)	5 (7.6%)
Clopidogrel	1 (1.2%)	1 (1.5%)
ACEI	24 (28.9%)	20 (30.3%)
Angiotensin receptor blockers	29 (34.9%)	25 (37.9%)
Beta blockers	60 (72.3%)	50 (75.8%)
Calcium blocker	12 (14.5%)	10 (15.2%)
Antiarrhythmic	8 (9.6%)	5 (7.6%)
Digitalis	9 (10.8%)	2 (3%)
Diuretics	39 (47%)	35 (53%)
Statins	55 (66.3%)	46 (69.7%)
SSRI	5 (6%)	5 (7.6%)

*ACEI* angiotensin-converting enzyme inhibitors, *CGNT* a composite score composed of the average of the *z*-scores of the following computer-generated neuropsychological tests: simple reaction time, sustained, selective, and divided attention. *MMSE* Mini-Mental State Exam, *MoCA* Montreal Cognitive Assessment, *NTB* composite neuropsychological test battery consisting of the average of the *z*-scores for Boston naming test, semantics verbal fluency, phonemic verbal fluency, trail making tests, clock-drawing test, and digit symbol substitution test; *SSRI* selective serotonin reuptake inhibitor

### Primary outcomes

Mean changes from baseline in each group, reported as least-square means (± SE), between-group differences with 95% confidence intervals, respective (unadjusted) *P*-values, and Cohen’s *d* effect sizes for between-group differences are shown in Table 2.

**Table 2:** Mean changes from baseline in dabigatran and warfarin groups for the primary cognitive outcomes. Data report the marginal effects (least-squares mean change from baseline score) adjusted for age (in years), log of years of education, and raw baseline score (mITT population, no imputation). Contrast values are between-group differences in the least-square mean change (dabigatran–warfarin). A positive value of contrast indicates a relative improvement (or smaller cognitive decline) of the group treated with dabigatran. There was no correction for multiple testing. Cohen’s *d* shows the effect size (contrast) as a proportion of the variation (residual standard deviation) of the adjusted least-square mean change

Cognitive assessment	Dabigatran		Warfarin		Difference (D-W) (95% CI)	*P*-value	Cohen’s *d* effect size
	Mean		Mean				
	(SD)	*N*	(SD)	*N*			
MMSE score	− 0.69 (− 1.18 to − 0.20)	83	− 0.57 (− 1.12 to − 0.01)	66	− 0.12 (− 0.88 to 0.63)	0.75	− 0.06
MoCA score	− 0.39 (− 0.94 to 0.16)	83	0.58 (− 0.04 to 1.19)	66	− 0.96 (− 1.80 to − 0.13)	0.02	− 0.39
NTB score	0.02 (− 0.06 to 0.10)	83	− 0.03 (− 0.12 to 0.06)	66	0.05 (− 0.07 to 0.18)	0.40	0.14
CGNT score							
Composite score	− 0.06 (− 0.16 to 0.04)	68	0.09 (− 0.02 to 0.20)	56	− 0.15 (− 0.30 to 0.006)	0.06	− 0.36
Simple reaction time (AC)	0.33 (0.008 to 0.64)	74	0.76 (0.40 to 1.11)	60	− 0.43 (− 0.92 to 0.05)	0.08	− 0.32
Simple reaction time (RT)	5.86 (− 62 to 73)	74	− 168 (− 244 to − 93)	60	174 (71 to 277)	0.001	0.60
Sustained attention (AC)	− 1.40 (− 3.00 to 0.25)	75	− 1.00 (− 2.90 to 0.88)	58	− 0.39 (− 2.94 to 2.15)	0.76	− 0.06
Sustained attention (RT)	36 (− 28 to 99)	75	− 28 (− 100 to 45)	58	63 (− 35 to 161)	0.21	0.23
Selective attention (AC)	− 0.35 (− 4.80 to 4.13)	75	2.80 (− 2.20 to 7.90)	59	− 3.20 (− 10.10 to 3.78)	0.37	− 0.17
Selective attention (RT)	69 (− 106 to 245)	75	− 189 (− 388 to 11)	59	258 (− 15 to 531)	0.06	0.34
Divided attention (AC)	1.47 (0.29 to 2.65)	72	0.13 (− 1.18 to 1.43)	59	1.35 (− 0.46 to 3.15)	0.14	0.27
Divided attention (RT)	3.50 (− 55 to 62)	72	− 17 (− 82 to 48)	59	20.80 (− 69 to 111)	0.65	0.09

*AC* accuracy, *CGNT* a composite score composed of the average of the *Z*-scores of the following computer-generated neuropsychological tests: simple reaction time, sustained attention, selective attention, and divided attention, *MMSE* Mini-Mental State Exam, *MoCA* Montreal Cognitive Assessment, *NTB* composite neuropsychological test battery consisting of the average of the *Z*-scores for Boston naming test, semantics verbal fluency, phonemic verbal fluency, trail making tests, clock-drawing test, and digit symbol substitution test, *RT* reaction time

**Fig. 2:**
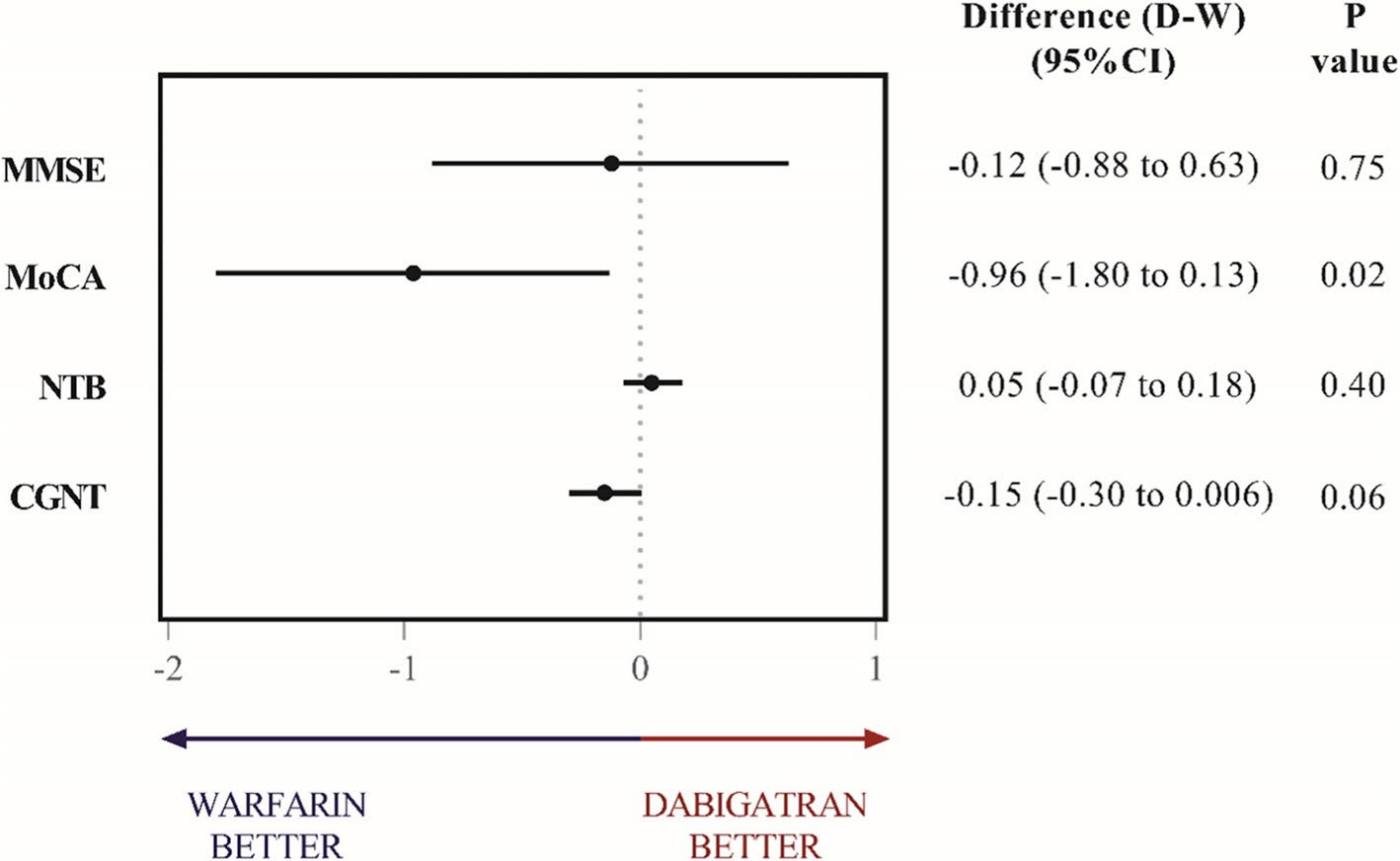
Primary cognitive outcomes in dabigatran and warfarin groups. There were no significant differences between dabigatran and warfarin treatment groups for most of the cognitive tests at 2 years (except for MoCA) in comparison to baseline. Comparison between D and W groups for the four tests that represent the primary cognitive outcomes. Differences between groups (95% CI) are expressed in the adjusted mean change from baseline (points) and 95% confidence intervals (segments) for the four primary outcomes

After controlling for age (in years), log of years of education, and raw baseline score, the difference between the mean change from baseline at 24 months in the dabigatran group minus warfarin group was not statistically significant for the MMSE, NTB, and CGNT scores. For CGNT, accuracies and reaction times of visual attention tests also failed to show a significant difference between the two study groups. For the MoCA score, we observed a significant difference when no correction for multiple testing is performed, suggesting less cognitive decline in the warfarin group. Figure 2 depicts the adjusted mean changes between groups from baseline estimates (points) and 95% confidence intervals (segments) for the four primary outcomes. Using Hommel’s correction for multiple comparisons, we obtained adjusted *P*-values of 0.74 for MMSE, 0.08 for MoCA, 0.74 for NTB, and 0.66 for CGNT, showing that detected between-group differences are not statistically significant.

### Exploratory outcomes

#### Cognitive decline per domain

**Fig. 3:**
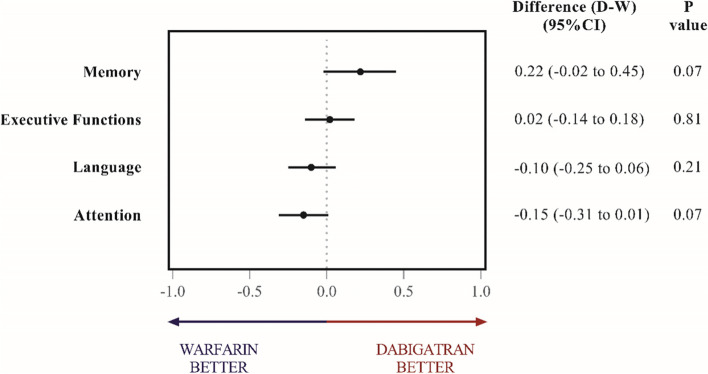
Exploratory cognitive outcomes in dabigatran and warfarin groups grouped by cognitive domains. Differences between dabigatran and warfarin treatment groups are depicted from baseline estimates (points) and 95% confidence intervals (segments) in the outcomes grouped by cognitive domains

There were no significant differences between dabigatran and warfarin treatment groups for all cognitive domains at 2 years in comparison to baseline (Fig. 3). No patient was diagnosed with dementia during the study.

#### Cognitive decline per anticoagulation quality in the warfarin group

Time in therapeutic range (TTR) in the warfarin group was 69.9% (± 13.9). In a post hoc analysis, no significant interaction was seen for the primary outcomes in the subgroup with TTR (≥ 70%), as shown in Additional file: Table S3.

There were 14 deaths during the study (five deaths in the dabigatran group and nine deaths in the warfarin group, *P* = 0.61). Among these deaths, seven were non-cardiovascular deaths (three in the dabigatran and four in the warfarin group, respectively) and seven CV deaths (two in dabigatran and five in warfarin group, respectively). Deaths were confirmed by death certificates and the cause of death were investigator-reported. We observed one episode of transient ischemic attack (TIA) and one stroke in patients from the Warfarin group. Four patients developed intolerance for dabigatran and one for warfarin (Fig. 1) and were excluded from the analyses.

## Discussion

GIRAF is the first randomized prospective and controlled trial comparing anticoagulant strategies in patients with AF or atrial flutter at risk of cognitive decline. The results of the analyses of the mean change from baseline in the MMSE, MoCA, NTB, and CGNT scores did not support our hypothesis that dabigatran would attenuate cognitive decline compared to warfarin, as no evidence of a beneficial effect of dabigatran was found between groups.

An extensive 90-min neuropsychological evaluation protocol with different tests was designed for the GIRAF trial to capture minimal differences in cognitive function between groups over time. The full trial protocol is available in the supplementary appendix. The use of a comprehensive range of tests grants to the GIRAF study a unique characteristic that distinguish it from the previous AF clinical trials evaluating cognitive function. The evaluation includes tests of global cognitive evaluation (MMSE, MoCA and NTB) and tests for specific cognitive domains, including the CGNT battery.

The exclusion of patients with previous stroke or dementia after baseline cognitive evaluation aimed to mitigate consequences of events before randomization. Although there was a significantly difference favoring the warfarin group in the MoCA score at 24 months, that was not confirmed in the more exhaustive and comprehensive cognitive tests (NTB, CGNT) nor in the exploratory outcomes of cognitive domains (memory, executive function, language, and attention). Therefore, caution should be used when interpreting the results of the MoCA score separately.

Through more stable and predictable pharmacokinetics [24], dabigatran could be more effective to prevent than VKAs to prevent cognitive impairment by reducing both thrombus formation/cerebral micro-embolism and cerebral microhemorrhage. Several prior observational studies suggested that AF patients receiving non-vitamin K oral anticoagulants were less likely to be diagnosed dementia [12, 13] or the combination of stroke, TIA, and dementia, compared to VKAs users. Other studies, however, showed similar risks of dementia with warfarin and non-vitamin K oral anticoagulants [7, 11]. These conflicting results might be explained by inherent limitations to study design, such as residual confounding, unknown baseline cognitive status, misclassification, and stopping/switching oral anticoagulants during the follow-up period. Few studies [11] provided a direct comparison between VKA and non-vitamin K oral anticoagulants on the risk of specific subtypes of dementia (e.g., vascular dementia and Alzheimer´s disease) and understanding of the mechanisms behind cognitive protection from non-vitamin K oral anticoagulants are largely putative. Our cognitive domain analysis, evaluating the relative impactive of dabigatran and warfarin in memory, executive function, language, and attention, was designed to provide key information to address this knowledge gap. We observed no significant differences between study groups in studied cognitive domains.

The lack of benefit from dabigatran in our trial might be related to a very well-managed warfarin administration. GIRAF trial patients randomized to warfarin had a TTR of roughly 70%, higher than in previous pivotal studies of non-vitamin K oral anticoagulants [10] and strikingly divergent from real-data in anticoagulation quality [25], especially in low and middle-income countries [26–28], with TTR levels as low as 23%. Also, even in patients with adequate TTR, stability over longer periods is unknown [29]. Indeed, observational studies suggests an association of warfarin therapy quality and cognition: both poor control [30] and supra-therapeutic [31] INRs are associated of an increased risk of dementia.

Cerebral hypoperfusion, inflammation, and AF-induced neuroendocrine disturbances are also proposed mechanisms [3, 32] underlying the increased risk of dementia in patients with AF that were not addressed in the GIRAF trial. Ongoing randomized clinical trials, evaluating the effects of different interventions on cognitive function [32] in patients with AF, will also support an in-depth understanding of this complex interaction between putative mechanisms and cognitive dysfunction.

Our study has important limitations. First, fewer patients in the warfarin group completed the 24-month cognitive assessment, due to an increased dropout rate, which could have biased the treatment effect. However, because we considered only patients with cognitive evaluation at baseline and 24 months in the mITT analysis with the primary outcome being the difference within each patient for the cognitive tests, used a linear model for the analysis of the co-primary outcomes, and performed two sensitivity analyses that were consistent with our main findings, we do not believe that our high attrition rate affected the observed differences between study arms. In addition, prior trials [33–35] testing interventions for cognitive decline had similar attrition rates.

Second, since cognitive decline was lower than expected in the warfarin group and the expected size effect of dabigatran in attenuating cognitive decline was not observed, the trial was underpowered to show a between-treatment difference. The very adequate anticoagulation regimen with warfarin in the GIRAF trial (TTR of 70%) could have an effect, protecting patients against greater cognitive decline.

Third, we included mainly patients with low educational level, a known risk factor for dementia in early life [8], and these results should not be extrapolated to other populations. Fourth, as we had very strict inclusion and exclusion criteria, the impact of different anticoagulant strategies in subgroups of patients that were excluded according to GIRAF trial design (such as patients with valvular heart disease) is not determined by our findings.

Finally, we cannot exclude that a 24-month window for cognitive evaluation was inadequate to examine if dabigatran would have a favorable effect in cognition, and studies with extended follow-up periods are warranted. Notably, despite analyzing only Alzheimer’s disease patients, prior randomized trials were able to demonstrate an intervention effect in cognition after 24 months [19, 35].

The GIRAF trial has also several strengths: the extensive and thorough cognitive evaluation to assess global cognitive performance and different cognitive domains and a prospective evaluation of cognition in a randomized controlled trial. Although a first step has been made in how to measure cognitive function in patients with AF [31], there is no gold standard for the ideal combination of tests that should be selected in randomized clinical trials. We believe that the GIRAF trial helps the pace of progress, as the NTB test selection and innovative CGNT can provide an acceptable standard for future trials.

## Conclusions

In conclusion, for elderly patients with atrial fibrillation, and without cognitive impairment at baseline, who did not have stroke and are adequately treated with warfarin (TTR of 70%) or dabigatran for 2 years, there was no difference in most of the cognitive outcomes. As GIRAF is hypothesis generation trial that adopted unique methods for cognitive evaluation, these findings could sow the seeds of future exploration and research in this area.

## Supplementary Information

**Additional file 1: Table S3**. Mean change from baseline according to TTR subgroups (< 70% and ≥ 70%) in the warfarin group. **Table S4**. Missing value percentages for each score regarding baseline and post-treatment test. **Table S5**. Missing value percentages for each score regarding baseline and post-treatment test scores. **Table S6**. Pooled descriptive statistics (Median [IQR]) for the post-treatment score values in the original and first three imputed data sets. **Tables S7-10**. Pooled results (using Rubin’s rule) for the linear regression analyses over the 10 imputed datasets for each score. **Table S11**. Pooled results (using Rubin’s rule) for the linear regression analyses over the 10 imputed datasets for each score. **Table S12**. Adjusted p-values for the regression analyses of the group effect by using Holm’s (1979) and Hommel’s (1988) formulas, as provided in the R stats package. **Figure S4**. Distributions of Age, log Education and Baseline scores for the groups with observed and missing values of the post-treatment MMSE score. **Figure S5**. Distributions of Age, log Education and Baseline scores for the groups with observed and missing values of the post-treatment MMSE score. **Figure S6**. The plots below refer to the NTB score. **Figure S7**. The plots below show distribution of Age and Log Education segmented by missingness of baseline CGNT. **Figure S8**. Histograms for Age, Log Education and Baseline score values according to missingness of post-treatment values. **Figure S9**. Estimate and 95% confidence intervals for the contrasts (W-D) for each score. **Figure S10**. Repeated analysis while performing imputation separately in each group.

**Additional file 2.** CONSORT Checklist.

## Data Availability

The datasets used and/or analyzed during the current study are available from the corresponding author on reasonable request.

## Abbreviations

GIRAF: Cognitive Impairment Related to Atrial Fibrillation Prevention Trial
MoCA: Montreal Cognitive Assessment
MMSE: Mini-Mental State Exam
NTB: Composite neuropsychological test battery
CGNT: Computer-generated tests
CI: Confidence interval
TTR: Time in therapeutic range
AF: Atrial fibrillation
VKA: Vitamin K anticoagulants
RCTs: Research clinical trials
RE-LY trial: The Randomized Evaluation of Long-Term Anticoagulation Therapy
ECG: Electrocardiogram
CHA_2_DS_2_-VASc: Score for the risk of systemic embolization
KDIGO: Chronic kidney disease grade
REDCap: Research Electronic Data Capture
INR: International normalized ratio
eGFR: Estimated glomerular filtration rate
BNT: Boston naming test
CDT: Clock drawing test
DSST: Digit symbol substitution test
FAS: Phonemic verbal fluency test
SVF: Semantic verbal fluency test
mITT: Modified intention-to-treat
D: Dabigatran
W: Warfarin
SE: Standard error
TIA: Transient ischemic attack
