# Supplementary material for: Effects of dabigatran versus warfarin on 2-year cognitive outcomes in old patients with atrial fibrillation: results from the GIRAF randomized clinical trial
Source: BMC Med. 2022 Oct 26;20:374. doi: 10.1186/s12916-022-02563-2 (PMC9598018; doi:10.1186/s12916-022-02563-2)
Supplement: Supplementary file 1 — Additional file 1: Table S3. Mean change from baseline according to TTR subgroups (< 70% and ≥ 70%) in the warfarin group. Table S4. Missing value percentages for each score regarding baseline and post-treatment test. Table S5. Missing value percentages for each score regarding baseline and post-treatment test scores. Table S6. Pooled descriptive statistics (Median [IQR]) for the post-treatment score values in the original and first three imputed data sets. Tables S7-10. Pooled results (using Rubin’s rule) for the linear regression analyses over the 10 imputed datasets for each score. Table S11. Pooled results (using Rubin’s rule) for the linear regression analyses over the 10 imputed datasets for each score. Table S12. Adjusted p-values for the regression analyses of the group effect by using Holm’s (1979) and Hommel’s (1988) formulas, as provided in the R stats package. Figure S4. Distributions of Age, log Education and Baseline scores for the groups with observed and missing values of the post-treatment MMSE score. Figure S5. Distributions of Age, log Education and Baseline scores for the groups with observed and missing values of the post-treatment MMSE score. Figure S6. The plots below refer to the NTB score. Figure S7. The plots below show distribution of Age and Log Education segmented by missingness of baseline CGNT. Figure S8. Histograms for Age, Log Education and Baseline score values according to missingness of post-treatment values. Figure S9. Estimate and 95% confidence intervals for the contrasts (W-D) for each score. Figure S10. Repeated analysis while performing imputation separately in each group. [file 12916_2022_2563_MOESM1_ESM.docx]

Supplementary Appendix

Approved final version of the protocol…………………………………………..………...1

Cognitive Assessment Protocol…………………………………………………………….7

Table S3. Mean change from baseline according to TTR subgroups (< 70% and ≥ 70%) in the warfarin group.…………………………………………………………………………….47

Additional statistical analysis……………………………………………………………...49

**Approved final version of the protocol**

Cognitive Impairment in Patients with Atrial Fibrillation (GIRAF Study)

*CoGnitive Impairment Related to Atrial Fibrillation (GIRAF) Prevention Trial*

Principal Investigator (PI) and Performer: Prof. Bruno Caramelli1 Coordinating Center: *Instituto do Coração(InCor)HCFMUSP*

*Participating centers: Federal University of Minas Gerais(UFMG), HCFMG*

*Performing Unit: Clinical Unit of Interdisciplinary Medicine in Cardiology, Heart Institute (InCor), Department of Cardiopneumology, FMUSP*

*Sponsorship:* Boehringer Ingelheim International GMBH

Abstract Introduction. Atrial fibrillation (AF) is the most common type of cardiac arrhythmia and is associated with cognitive decline and dementia. Cognitive and functional decline have been neglected in clinical trials conducted on patients with AF. Dabigatran is a new drug that may offer more stable anticoagulant therapy over time compared to warfarin. This advantage could be associated with a low incidence of cognitive and functional decline.

Objective. To evaluate the effect of dabigatran compared to warfarin on cognitive and functional decline, thrombin generation, the occurrence of hemorrhage, and cerebrovascular complications in elderly patients with AF.
Methods. We will evaluate 200 patients with AF who will be randomized to receive dabigatran, 150 mg twice daily or warfarin, once daily, to maintain the international normalized ratio (INR) between 2 and 3. After 1 year and at the end of the study at 2 years, paticipants will be assessed for cognitive outcomes. In addition, patients will undergo two magnetic resonance imaging brain scans at the beginning and end of the study to detect possible cerebrovascular complications.

Hypothesis. The cognitive and functional decline observed in patients with AF is related to thrombotic and/or cardioembolic events. Regarding the outcomes of cognitive and functional decline, warfarin is less efficient than dabigatran, which is an anticoagulant with a stable action profile. For this reason, dabigatran offers protection against thrombotic phenomena and, consequently, attenuates the process of cognitive and functional decline related to AF.

Expected results and implications. If the expected results are confirmed, the standard treatment for the prevention of AF will have been completely modified and expanded with the insertion of objectives related to cognitive preservation to benefit patients and their families.

1. Introduction

Atrial fibrillation (AF) is the most common type of cardiac arrhythmia, with its incidence increasing with age. Cerebrovascular complications represent a major challenge and have been intensively evaluated in clinical trials with AF patients. Indeed, the prevention of ischemic stroke is the most common and studied outcome in clinical trials with AF patients. Cognitive and functional decline, on the contrary, has been partially neglected in these studies. A recent *post hoc* analysis included 31,546 patients with AF and concluded that even in the absence of stroke, cognitive and functional decline were associated with AF at long-term follow-up. Furthermore, another recent meta-analysis concluded that AF is independently associated with a risk of dementia. In the current scenario of a global aging of the population, both the quantity and quality of life are equally important goals, with cognitive and functional preservation being most valuable.

The standard treatment for the prevention of neurovascular complications of AF is anticoagulation with warfarin. On the contrary, treatment with warfarin is associated with some difficulties, such as a narrow therapeutic margin, that is, the interval between the ineffective and toxic doses, which is associated with severe hemorrhagic phenomena. Thus, these medications require strict monitoring with periodic coagulation tests (prothrombin time). Moreover, an interaction with several other drugs and types of food and alcohol occurs. Perhaps, for this reason, patients with AF on treatment with warfarin are undertreated or spend a significant percentage of the time taking the medications outside their therapeutic range. Furthermore, in the long term, warfarin, which has been available for over 60 years, has proven effective and safe in preventing neurovascular complications in several subgroups of patients with AF.

Recently, new anticoagulant drugs have been developed and shown to be non-inferior to warfarin for the prevention of stroke. These drugs have additional advantages over warfarin because they offer stable anticoagulant therapy over time and do not show interaction with food and alcohol. This high stability could be responsible for offering patients with AF long periods of effective anticoagulation. This advantage cannot be identified by assessing major outcomes, such as stroke but can be evidenced by assessing equally important outcomes, such as cognitive and functional decline, which, unfortunately, have not been analyzed so far.

2. Objectives

Primary:
- To evaluate the effect of dabigatran compared to warfarin on cognitive and functional decline, as a result of the disease itself or its progression, in elderly patients with AF.
Secondary:
- To evaluate the effect of dabigatran compared to warfarin on thrombin generation in elderly patients with AF.

- To evaluate the effect of dabigatran compared with warfarin on the occurrence of hemorrhage in elderly patients with AF.

- To evaluate the effect of dabigatran compared to warfarin on the prevention of transient ischemic attack (TIA) and/or stroke in elderly patients with AF.

2.1 Hypothesis

The cognitive and functional decline observed in patients with AF is related to thrombotic and/or cardioembolic events. Regarding the outcomes of cognitive and functional decline, warfarin is less efficient than dabigatran, which is an anticoagulant with a stable action profile. For this reason, dabigatran offers protection against thrombotic phenomena and, consequently, attenuates the process of cognitive and functional decline related to AF.

3. Methodology

*3.1 Delineation and population*

The GIRAF study is a randomized, prospective, and parallel clinical trial blinded to the interpretation of outcomes. It will include 200 patients in three centers, two of which are in São Paulo, at the Hospital das Clinicas, School of Medicine, USP, which is the coordinating center at Prevent Senior and a co-participating center. Another center is in Minas Gerais, at the Hospital de Clínicas, School of Medicine, UFMG, which is the participating center. Patients with AF will be included according to the following criteria:

*3.2 Inclusion criteria*

1. Diagnosis of AF confirmed (and recorded) by one of the three criteria below:

A. Electrocardiography (ECG) on the day of the first evaluation or randomization.

B. The patient had a symptomatic episode of paroxysmal or persistent AF documented (and recorded) by ECG up to 6 months before randomization.

C. Documentation compatible with paroxysmal or persistent AF, symptomatic or asymptomatic, on two separate occasions, for at least 1 day, with one of them less than 6 months from randomization. In this case, AF can be documented by ECG, a rhythm strip (monitor), pacemaker evaluation, an implantable defibrillator, or a Holter monitor. The duration of the AF episode should be at least 30 seconds. However, electrograms obtained from pacemaker or implantable defibrillators may represent only one of the required occasions.

2. Age over 70 years

3. Both sexes

4. CHA2DS2-VASc score > 1

5. Signed the informed consent form (ICF)

3.3 Exclusion criteria

1. Valvular heart disease (prosthesis or clinically relevant disease), prior stroke, or TIA.

2. Dementia or other severe neurological condition.

3. Major surgery within the last 30 days.

4. Surgery planned within the next 3 months.

5. Intracranial, intraocular, spinal, retroperitoneal, or intra-articular bleeding in the absence of trauma.

6. Gastrointestinal bleeding within the past 12 months.

7. Symptomatic or endoscopically detected gastric ulcer within the past 30 days.

8. Illness or hemorrhagic diathesis.

9. Use of fibrinolytic agents within the past 48 hours.

10. Uncontrolled hypertension (systolic blood pressure > 180 and/or diastolic blood pressure > 100).

11. Active cancer or radiotherapy in the last 6 months, with the expectation of survival less than 3 years.

12. Contraindication to the use of warfarin.

13. Reversible and circumstantial causes of AF (postoperative, heart surgery, pulmonary embolism, or hyperthyroidism).

14. Surgery for AF treatment (ablation) scheduled.

15. Creatinine clearance less than 30 ml/min.

16. Active infectious endocarditis.

17. Active liver disease defined by active hepatitis (A, B, or C) or transaminases levels at levels greater than twice the reference value.

18. Anemia (hemoglobin levels <10.0 mg%) or thrombocytopenia (count < 100,000).

19. Severely compromised ventricular function, defined by ejection fraction on echocardiogram lower than 35%.

20. Refusal to sign the ICF.

3.4 Procedures

Patients will be randomized to receive warfarin (once daily to maintain the international normalized ratio (INR) between 2 and 3) or dabigatran (150 mg twice daily, 110 mg twice daily for patients older than 80 years or those with creatinine clearance between 30 and 50 mL/min) for 2 years. For patients already on oral anticoagulants, controlled withdrawal (*wash-out)* will be performed before randomization as follows:

- For conversion from warfarin to dabigatran, start dabigatran when the INR is < 2.
- For conversion from dabigatran (or other oral anticoagulant) to warfarin, start warfarin according to the creatinine clearance: if ≥ 50 mL/min, start warfarin 3 days before discontinuing dabigatran or if between 30_–_50 mL/min, start warfarin 2 days before discontinuing dabigatran.

At inclusion after 1 year and at the end of the study at 2 years, the individuals will be evaluated for cognitive outcomes according to the National Institute of Neurological Disorders and Stroke-Canadian Stroke Network Vascular Cognitive Impairment Harmonization Standards. This standard recommends three assessment protocols (5, 30, and 60 minutes) with several tests, the main one being the Mini Mental State Examination (Appendix 1). For this study, we will use only the 60-minute protocol complemented by the Montreal Cognitive Assessment (MoCA) test. (Appendix 2). All assessments will be performed by personnel experienced in the application and interpretation of the tests. In addition, the patients will undergo two cerebral nuclear magnetic resonance exams

and a carotid ultrasound examination to search for atherosclerotic diseases at the beginning and end of the study. If the patient is unable to undergo the magnetic resonance imaging exam for some reason (for example: claustrophobia or the presence of a definitive pacemaker or a prosthesis), we will perform the skull computed tomography exam at the beginning and end of the study.

The cardiologic inclusion and exclusion criteria will be analyzed by experienced cardiologists who will follow the patients throughout the study period for clinical control, performing medical consultations every 3 months.

***3.5 Laboratory examinations***

The tumor necrosis factor-alpha levels using the enzyme-linked immunosorbent assay (ELISA) method (Thermo Scientific) will be analyzed only at the inclusion in the study.

For the comparative analysis of the changes promoted in blood coagulation according to the treatment, the following exams will be performed at the beginning, 30 days, 1 year, and 2 years after randomization:

- Thrombin generation by the calibrated automated thrombogram method by Hotia Medical;
- D-dimer levels by the immunoturbidimetry method (Liatest® D-Di Plus, diagnostics Stago) ;
- Fibrinopeptide A levels by ELISA (Zymutest FPA, Hyphen Biomed)

3.6. Sample size

To calculate the sample sizes, we considered the composite cognitive outcome (at least one) among all outcomes assessed by the National Institute of Neurological Disorders, Stroke-Canadian Stroke Network Vascular Cognitive Impairment Harmonization Standards and the MoCA test. Based on a previous study evaluating patients with AF and the potential benefit of dabigatran for its high therapeutic stability, we estimated the occurrence of cognitive outcomes to be 20% and 40% in the dabigatran and warfarin groups, respectively. Additionally, after a two-tailed test with a power of 80% and an α of 0.05, we reached the necessary number of 91 patients per group. Finally, considering eventual dropouts, it was decided that 100 patients will be included in each group.

4. Timeline


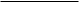


| WEEKEND | 1st /wk | 2nd /wk | 3rd /wk | 4th /wk | 5th /wk | 6th /wk |
| --- | --- | --- | --- | --- | --- | --- |
| Data collection and inclusion | x | x | x | x |  |  |
| Analysis of results - statistics |  |  |  |  | x | x |

Wk: week

*Will be validated after approval by the Ethics Committee

**Cognitive Assessment Protocol**

**Mini-Mental State Examination**

(Folstein et al., 1975; Brucki et al., 2003)

- ORIENTATION TO TIME: ________/5

- Day of the week, day of the month, month, year, approximate time

- ORIENTATION TO PLACE: __________/5

- Location, (hospital, residence, clinic), floor, street or neighbourhood, city, state

- IMMEDIATE MEMORY:__________/3

- Vase, car, brick

- ATTENTION AND CALCULATION:________/5

- 100 - 7 = ... (up to 65)

- EVOCATION:________/3

- Remember the 3 words

- LANGUAGE & CONSTRUCTIVE SKILLS

- Name a watch and a pen:______/2

- Repeat: ‘NEITHER HERE, NOT THERE, NOR OVER THERE.’:_______/1

- Command: ‘Take this paper with your right hand, fold it in half, and place it on the ground.’:________/3

- Read and comply: ‘CLOSE YOUR EYES.’:_______/1

- Write a sentence:______/1

- Copy a drawing:_______/1

SCORE_____ / 30

CLOSE YOUR EYES

**Mini-Mental State Examination - MMSE (Folstein et al., 1975; Brucki et al., 2003)**

**Orientation to time** - Ask the individual:

. *What day is it today?*

*. What month is it?*

*. What year is it?*

*. What day of the week is it?*

*. What is the approximate time*? (Accept a range of plus or minus one hour)

Give one point for each correct answer.

**Orientation to space** - Ask the individual:

. *Where are we? (office, dormitory, living room - pointing to the floor)*

*. What is this place? (pointing around in a broader sense: hospital, nursing home, own house).*

*. What neighbourhood are we in or what is the name of a nearby street?*

*. What city are we in?*

*. What state are we in?*

Give one point for each correct answer.

**Immediate memory**: *I will say 3 words and you will repeat them: car, vase, brick.*

Give 1 point for each word that is repeated correctly the first time, although it can be repeated up to 3 times for learning if there are mistakes. Use unrelated words.

**Calculation:** Subtraction of serial sevens (100-7, 93-7, 86-7, 79-7, 72-7, 65)

Give 1 point for each correct answer. If there is an error, correct it and proceed. Consider it correct if the examinee spontaneously corrects themselves.

**Word evocation:** Ask which words the subject has just repeated. Give 1 point for each.

**Naming:** Ask the subject to name the objects shown (clock, pen). Give 1 point for each.

**Repetition**: *Pay attention: I will tell you a sentence and I want you to repeat it after me: ‘Neither here, not there, nor over there.*’ Give 1 point only if the repetition is perfect.

**Command:** *Pick up this paper with your right hand* (1 point), *fold it in half* (1 point), *and place it on the ground* (1 point). Give a total of 3 points. If the subject asks for help while performing the task, do not give any hints.

**Reading:** Show the written sentence, CLOSE YOUR EYES, and ask the individual to do what is being commanded. Do not assist if they ask for help or simply read the sentence without carrying out the command (1 point).

**Sentence:** Ask the individual to write a sentence. If he/she does not understand the meaning, help with the following: a sentence that has a beginning, middle, and end; something that happened today; something he/she wants to say. No grammatical or spelling errors are considered (1 point).

**Copy a drawing:** Show the model and ask them to do the best they can. Consider only if there are 2 intersecting pentagons (10 angles) forming a figure with four sides or two angles (1 point).


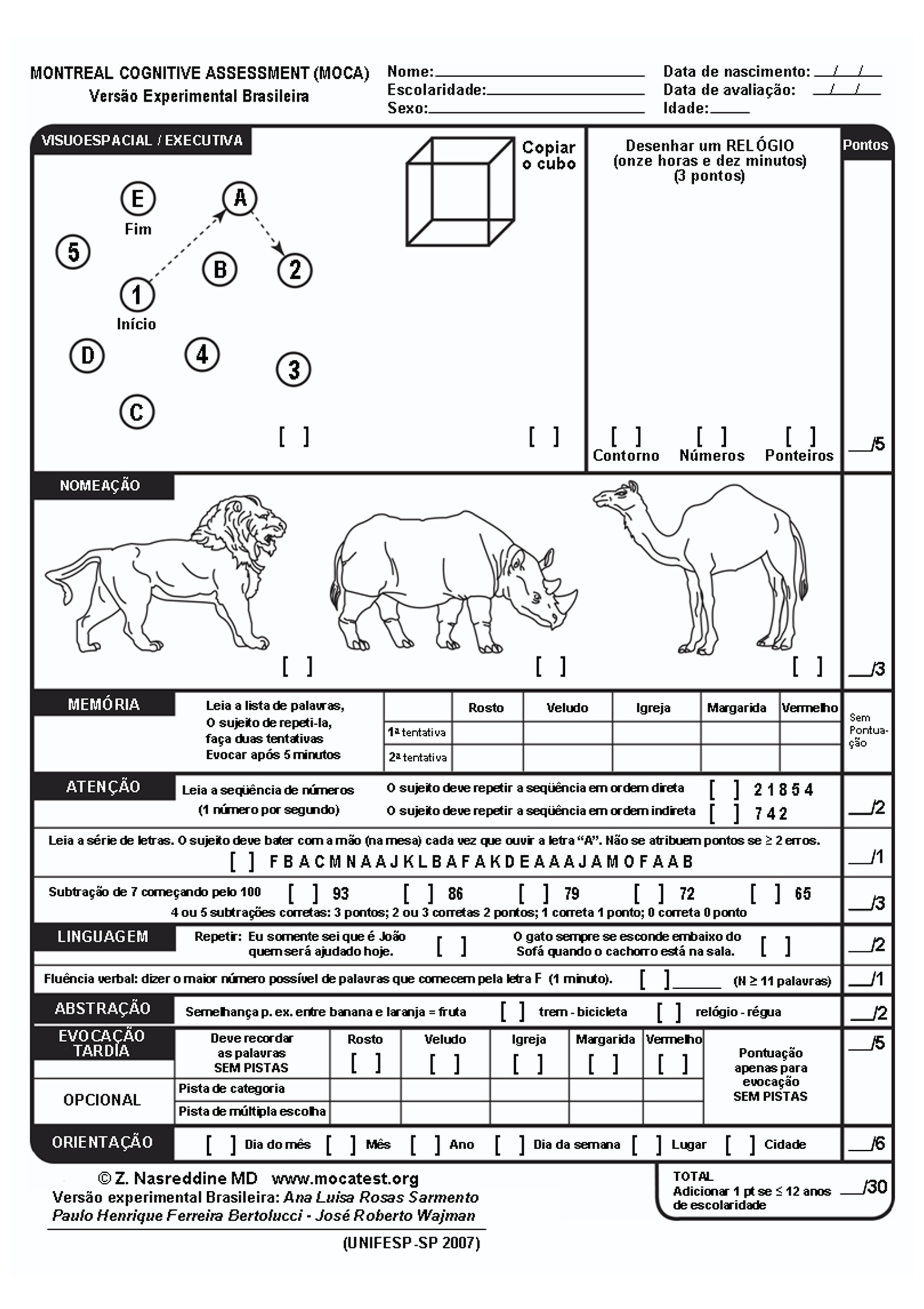


Montreal Cognitive Assessment (MoCA) Application and Scoring Instructions

The Montreal Cognitive Assessment (MoCA) was designed as a rapid screening instrument for mild cognitive dysfunction. It accesses different cognitive domains: attention and concentration, executive functions, memory, language, visuoconstructional skills, conceptual thinking, calculations, and orientation. Time to administer the MoCA is approximately 10 minutes. The total score is 30 points; a score of 26 or above is considered normal.

Computer generated Neuropsychological tests (CGNT)

All tests were designed on the E-Prime software as described on reference 16

Rodrigues AC, Lima MDM, de Souza LC, Furtado C, Marques CE, Gonçalves L, Lima MV, Lasmar RP, Caramelli P. No Evidence of Association Between Soccer Heading and Cognitive Performance in Professional Soccer Players: Cross-Sectional Results. Front Neurol. 2019 Mar 12;10:209

1. Alternating Trail Making

Administration: The examiner instructs the subject: *‘Please draw a line, going from a number to a letter in ascending order. Start here {point to (1)} and draw a line from 1 to A, then to 2, and so on. End here {point to (E)}.’*

Scoring: Allocate 1 point if the subject successfully draws the pattern 1- A-2-B-3-C-4-D-5-E, without drawing any lines that cross. Any error that is not immediately self-corrected earns a score of 0.

2. Visuoconstructional Skills (Cube)

Administration: The examiner gives the following instructions, pointing to the cube: *‘Copy this drawing as accurately as you can, in the space below’.*

Scoring: One point is allocated for a correctly executed drawing.

- Drawing must be three-dimensional.
- All lines are drawn.
- No line is added.
- The lines are relatively parallel, and their length is similar (rectangular prisms are accepted). A point is not assigned if any of the above criteria are not met.

3. Visuoconstructional Skills (Clock)

Administration: Indicate the right third of the space and give the following instructions: *‘Draw a clock. Put all the numbers on it and set the time to 10 after 11’.*

Scoring: One point is allocated for each of the following three criteria:

- Contour (1 point): The clock face must be a circle with only minor distortion acceptable (e.g. slight imperfection regarding closing the circle);
- Numbers (1 point): All clock numbers must be present, with no additional numbers; numbers must be in the correct order and placed in the approximate quadrants on the clock face; Roman numerals are acceptable; numbers may be placed outside the circle contour.
- Hands (1 point): There must be two hands jointly indicating the correct time; the hour hand must be clearly shorter than the minute hand; hands must be centred within the clock face with their junction close to the clock centre. A point is not assigned if any of the above criteria are not met.

4 . Naming

Administration: Starting at the left, point to each picture and say, ‘*Tell me the name of this animal*’.

Scoring: Points are given as shown for the following answers: (1) camel or dromedary, (2) lion, (3) rhinoceros or rhino.

5 . Memory

Administration: The examiner reads a list of words at a rate of one per second, giving the following instructions*: ‘This is a memory test. I will read you a list of words, which you must remember now and later on. Listen carefully. When I am through, tell me as many words as you can remember. It doesn’t matter in what order you say them’.* Mark a check in the allocated space for each word the subject produces on the first trial. When the subject indicates that they are finished (remembered all the words) or that they cannot remember any more words, read the list a second time with the following instructions*: ‘I'll read the same list a second time. Try to remember and tell me all the words you can, including words said the first time’.* Put a check in the allocated space for each word the subject recalls on the second trial. At the end of the second attempt, inform the subject that they will be asked to recall these words again by saying*, ‘I will ask you to recall these words again at the end of the test’.*

Scoring: No points are given for Trials 1 and 2.

6 . Attention

Forward Digit Span

Administration: Give the following instructions*: ‘I am going to say some numbers, and when I am through, repeat them to me exactly as I said them’.* Read the five number sequence at a rate of one digit per second.

Backward Digit Span

Administration: Give the following instructions*: ‘Now I am going to say some more numbers, but when I am through, you must repeat them to me in the backwards order’.* Read the three number sequence at a rate of one digit per second. Scoring: Allocate 1 point for each sequence repeated correctly (Note: The correct answer for the backwards trial is 2-4-7).

Vigilance

Administration: The examiner reads the list of letters at a rate of one per second, after giving the following instructions: *‘I am going to read a sequence of letters. Every time I say the letter A, tap your hand once. If I say a different letter, do not tap your hand’.* Scoring: Give a point if there is no more than one error (an error is a tap on the wrong letter or failure to tap when hearing the letter A).

Serial 7s

Administration: The examiner gives the following instructions: *‘Now I will ask you to count by subtracting seven from 100, and then, keep subtracting seven from your answer until I tell you to stop’.* Give this instruction twice if necessary.

Scoring: This item is scored 3 points. Give no points (0) for incorrect subtraction, 1 point for one correct subtraction, 2 points for 2-3 correct subtractions, and 3 points if the participant successfully makes 4-5 correct subtractions. Count each correct subtraction out of 7, beginning from 100. Each subtraction is evaluated independently, i.e. if the participant answers with an incorrect number but continues to correctly subtract 7 from it, give 1 point for each correct subtraction. For example, the participant may answer 92-85-78-71-64 where 92 is incorrect but all subsequent numbers are subtracted correctly. This is one error and the item should be given a score of 3.

7 . Sentence Repetition

Administration: The examiner gives the following instructions: *‘I am going to read a sentence to you. Repeat after me exactly as I say it: I only know that John is the one to help today’.* Following the response, say*, ‘Now I'm going to read another sentence. Repeat it after me exactly as I say it [pause]: The cat always hid under the couch when dogs were in the room’.*

Scoring: Allocate 1 point for each sentence that is repeated correctly . Repetition must be exact. Be alert for omissions (e.g. omitting ‘only’ or ‘always’) and substitutions/additions (e.g. ‘John is the one who helped today’).

8 . Verbal Fluency

Administration: The examiner gives the following instructions: *'Tell me as many words as you can think of that begin with a certain letter of the alphabet, which I will tell you in a moment. You can say any kind of word you want, except for proper names (like Bob or Boston), numbers, or words that begin with the same sounds but have a different suffix, for example, love, lover, loving. I will tell you to stop after one minute. Are you ready? [Pause] Now, tell me how many words you can think of that start with the letter F. [Time for 60 seconds]. Stop’.*

Scoring: Allocate 1 point if the subject generates 11 or more words in 60 seconds. Record the subject's response in the bottom or side margins.

9 . Abstraction

Administration: The examiner asks the subject to explain what each pair of words has in common, starting with the example: *‘Tell me how an orange and a banana are alike’.* If the subject answers in a concrete manner, then say the following only once: ‘*Tell* *me another way in which those items are alike’.* If the subject does not give the appropriate response (fruit), say, *‘Yes, and they are also both fruit’.* Do not give any additional instructions or clarification. Following the response, administer the second trial, saying, ‘*Now tell me* *how a ruler and a watch are alike*’. Do not give any additional instructions or prompts.

Scoring: Only the last two item pairs are scored. Give 1 point for each correctly answered item pair . The following answers are accepted: train-bicycle = means of transportation, means of travelling, you take trips in both; ruler-watch = measuring instruments, used for measuring. The following answers are not accepted: train-bicycle = they have wheels; ruler-watch = they have numbers.

10. Delayed Recall

Administration: The examiner gives the following instructions: ‘*I read some words to you earlier that I asked you to remember. Tell me as many of these words as you can remember’.* Make a check mark (√) for each of the words correctly recalled spontaneously without any cues in the allocated space. Scoring: Allocate 1 point for each word recalled freely without any cues.

Optional

Following the delayed free recall trial, prompt the subject with the semantic category cue provided below for any word not recalled. Make a check mark (√) in the allocated space. If the subject remembers the word with the help of the category or multiple choice clue, hint at all the unremembered words with the help of a category or multiple-choice cue. If the subject does not remember the word after the category cue, give them a multiple choice trial, using the following example instruction: ‘Which of the following words do you think it was, nose, face, or hand?’

Use the following category and/or multiple choice cues for each word, when appropriate:

FACE category cue: part of the body; multiple choice: nose, face, hand

VELVET category cue: type of fabric; multiple choice: denim, cotton, velvet

CHURCH category cue: type of building; multiple choice: church, school, hospital

DAISY category cue: type of flower; multiple choice: rose, daisy, tulip

RED category cue: a colour; multiple choice: red, blue, green

Scoring: No points are allocated for words recalled with a clue. A cue is used for clinical information purposes only, as it may give the test interpreter additional information about the type of memory disorder. For memory deficits with retrieval failure, performance can be improved with a clue. For memory deficits due to encoding failures, performance does not improve with a cue.

11 . Orientation

Administration: The examiner gives the following instructions: *‘Tell me the date today’.* If the subject does not give a complete answer, then prompt them accordingly by saying*: ‘Tell me the [year, month, exact date and day of the week]’.* Then say, *‘Now, tell me the name of this place, and which city it is in’.*

Scoring: Give 1 point for each item correctly answered. The subject must give the exact date and the exact place (name of hospital, clinic, office). No points are allocated if the subject makes an error of one day regarding the day and date.

Total Score: Sum all sub-scores listed on the right-hand side. Add 1 point for an individual who has 12 years or fewer of formal education, for a possible maximum of 30 points. A final total score of 26 and above is considered normal.

FAS

*‘I will tell you a letter of the alphabet and I want you to say as many words as possible that begin with this letter in one minute. You must not answer with proper names (e. g. names of people, cities, countries) or derivative words (e. g., friend, friends, friendly.). Say your answers as quickly as you can. Are you ready? You may begin!’*

The final score for each letter is the result of the sum of the number of words cited by the subject, with the exception of proper names and derived words. In case of repetition of a word already said and that may have a different meaning from the previous one, at the end of the test, the examiner should ask the subject about the meanings of the cited words. Words said more than once that have the same meaning are scored only once. The sum of the subtotals of the results for each of the three letters is the subject's final score on the test.

Trail-Making Test

**Trail-Making Test, Part B**


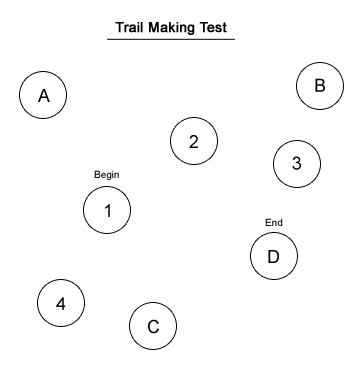


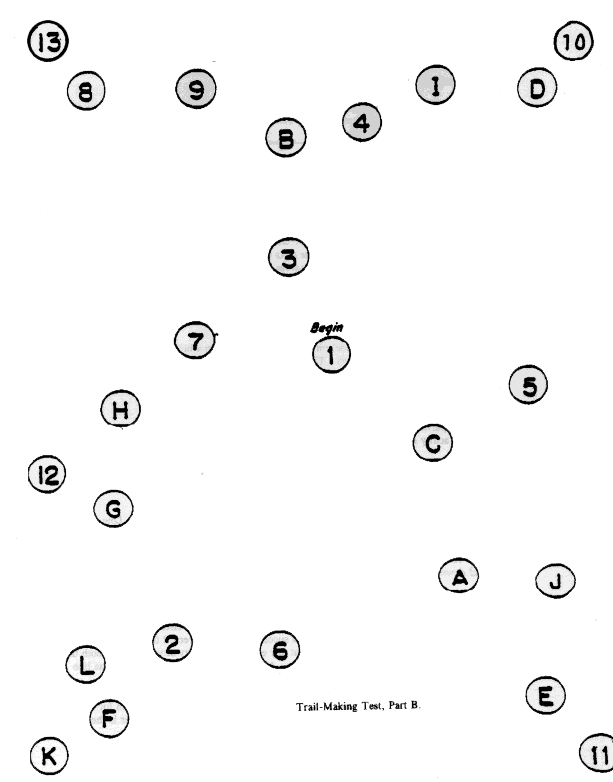


Measure execution time in seconds.

Boston Naming Test

- Say to the patient: ‘*I am going to show you a few pictures, your task is to tell me the name of the object shown in the pictures. If you don't know or can't remember the name, but you know what it is, tell me something about it’.*
- Write down all the answers exactly as the patient says them. The maximum presentation time for each drawing is 10 seconds.

INTERPRETATION:

Score < 13: abnormal (education level 8-9 years).

|  | **DRAWING** | **ANSWER** | **INCORRECT** | | **CORRECT** |
| --- | --- | --- | --- | --- | --- |
| High frequency | Tree |  | 0 | |   1   |
|  | Bed |  | 0 | | 1 |
|  | Whistle |  | 0 | |   1 |
|  | Flower |  | 0 | |   1 |
|  | House |  | 0 | |   1 |
| Medium frequency | Canoe |  | 0 | |   1 |
|  | Toothbrush |  | 0 | |   1   |
|  | Volcano |  | 0 | | 1 |
|  | Mask |  | 0 | |   1 |
|  | Camel |  | 0 | |   1 |
| Low frequency | Harmonica |  | 0 | |   1 |
|  | Ice tongs |  | 0 | |   1   |
|  | Hammock |  | 0 | | 1 |
|  | Funnel |  | 0 | |   1 |
|  | Dominoes |  | 0 | |   1   |
| PARTIAL SCORE | | High frequency: | Medium frequency: | Low frequency: | |


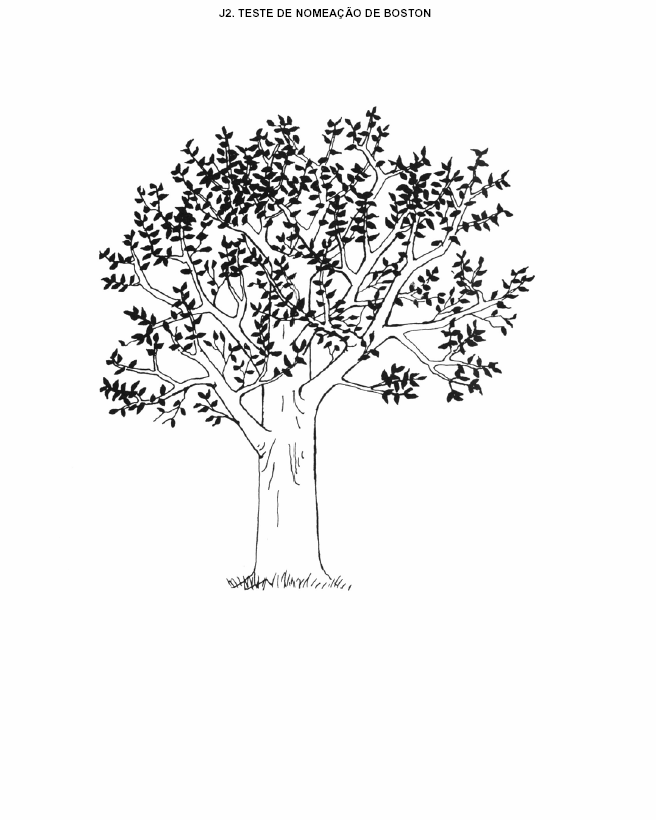


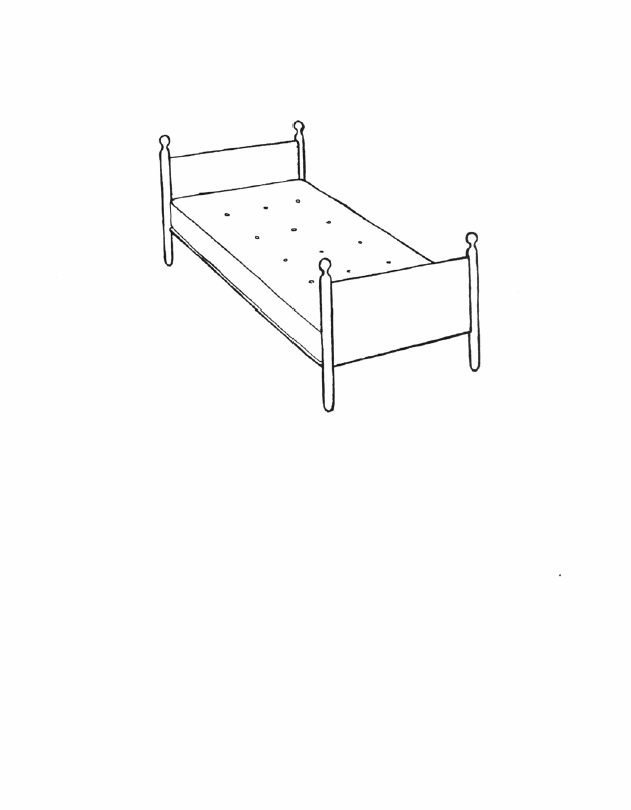


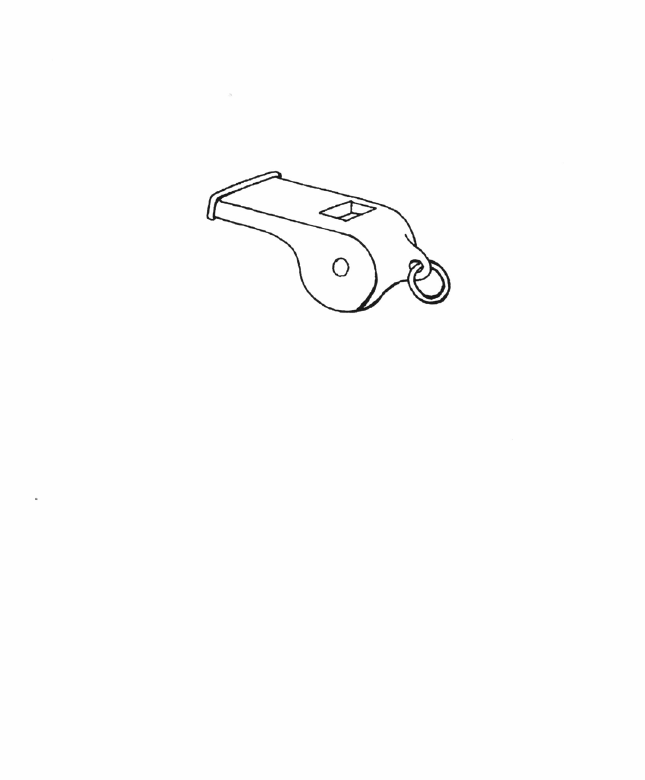


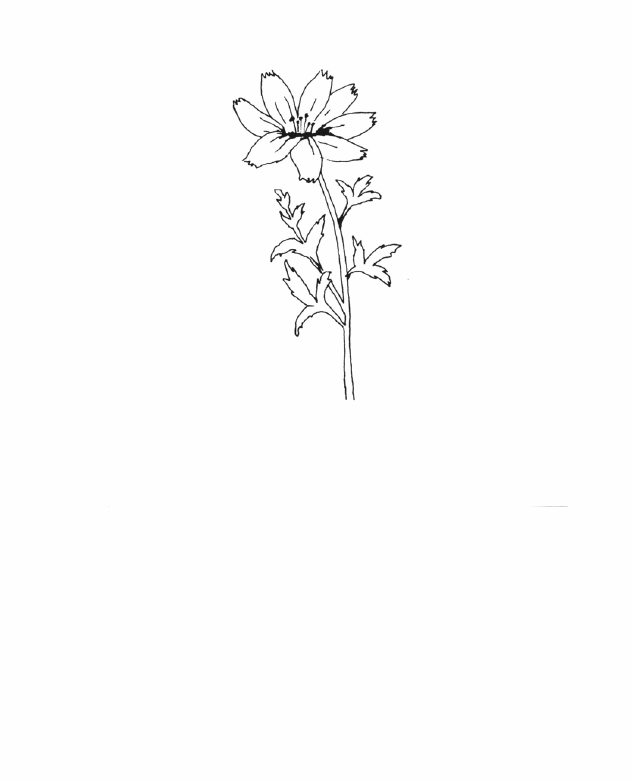


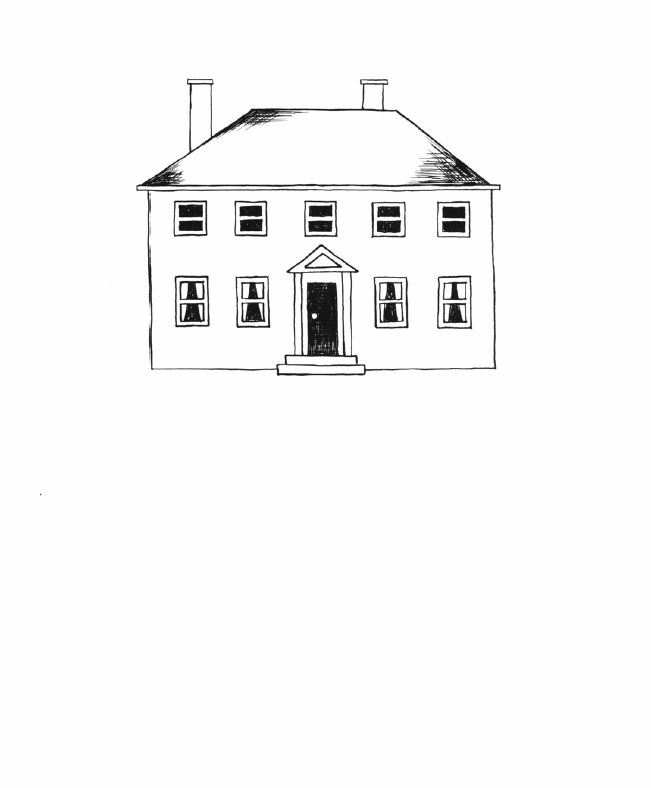


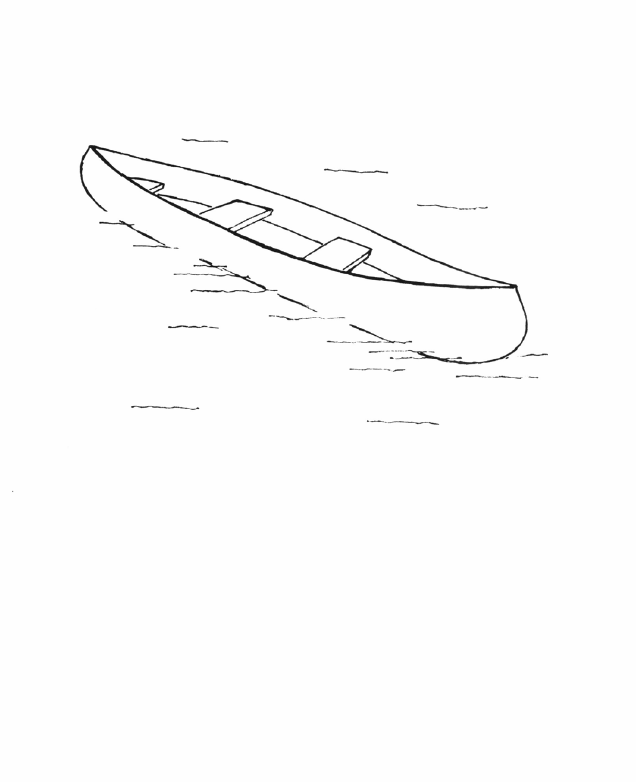


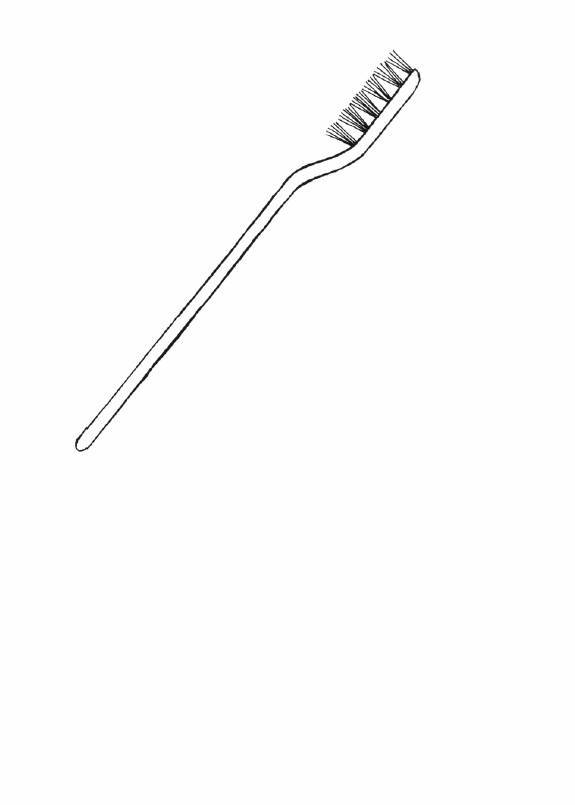


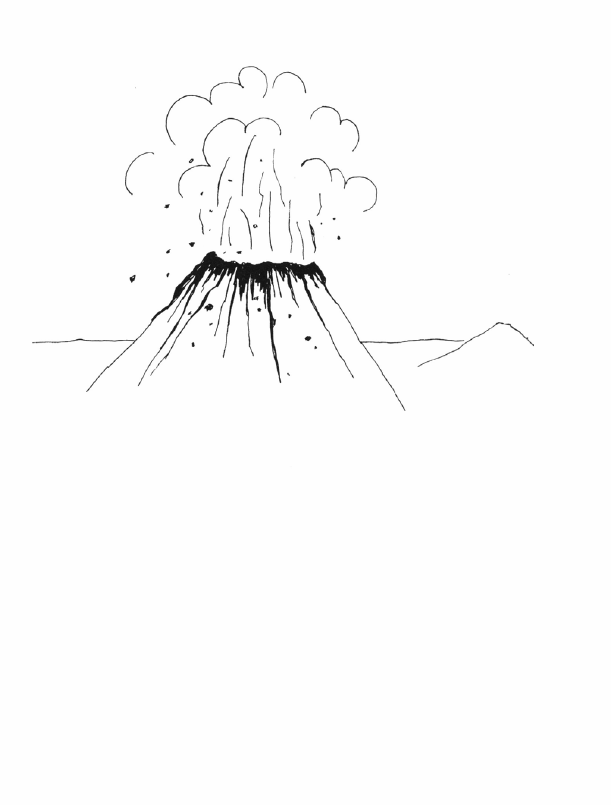


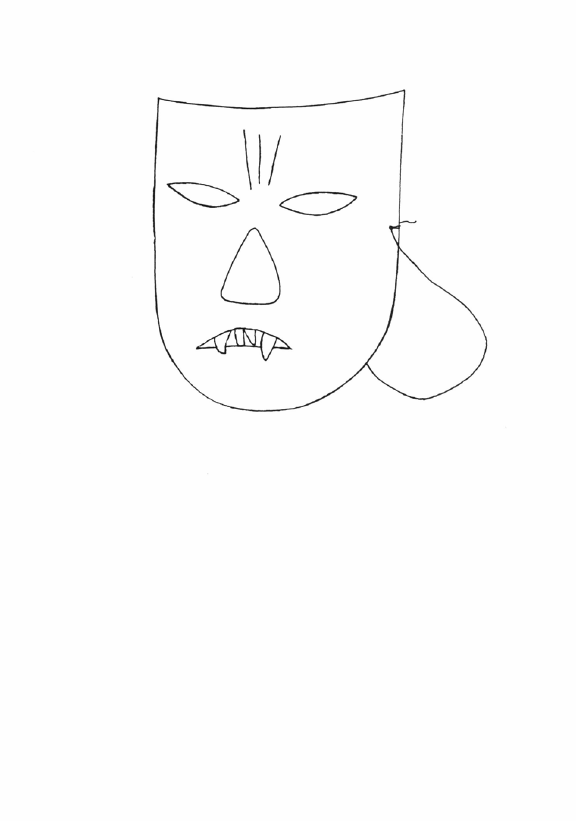


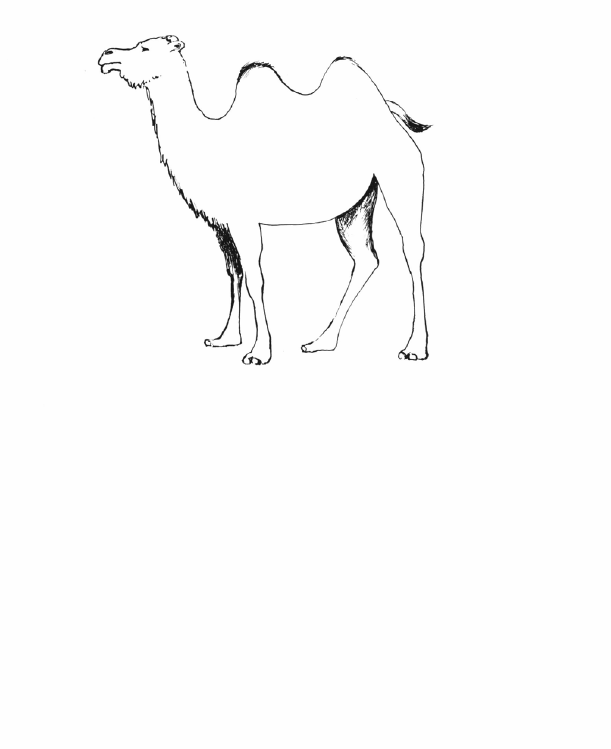


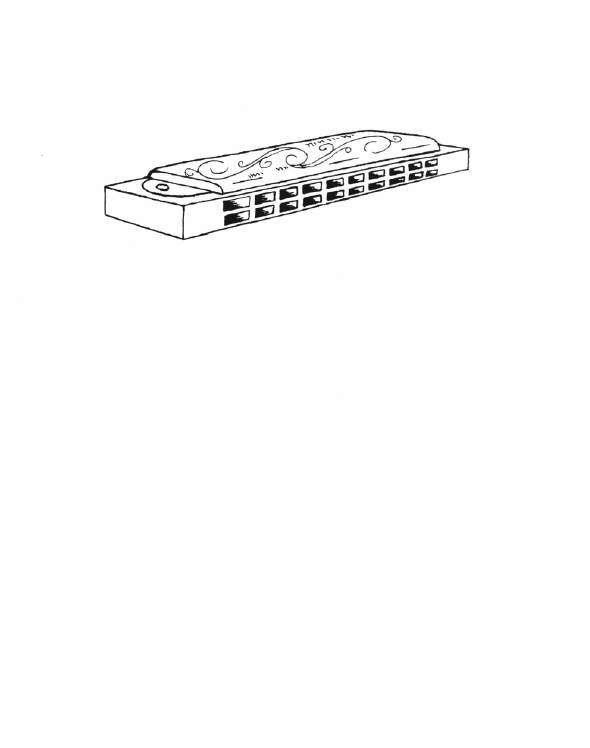


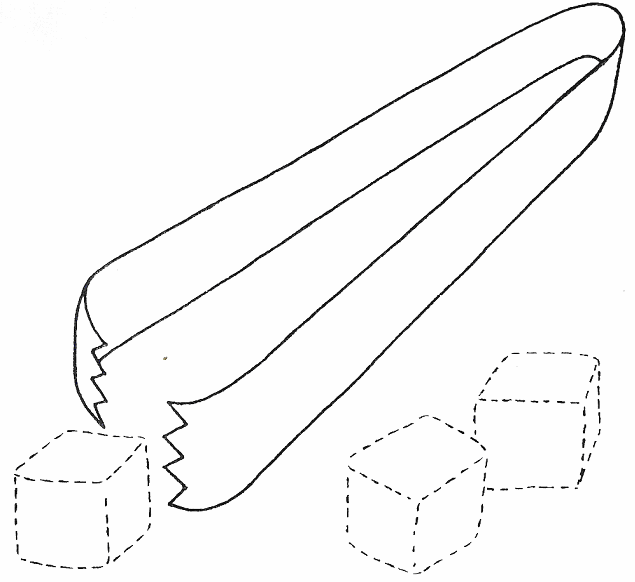


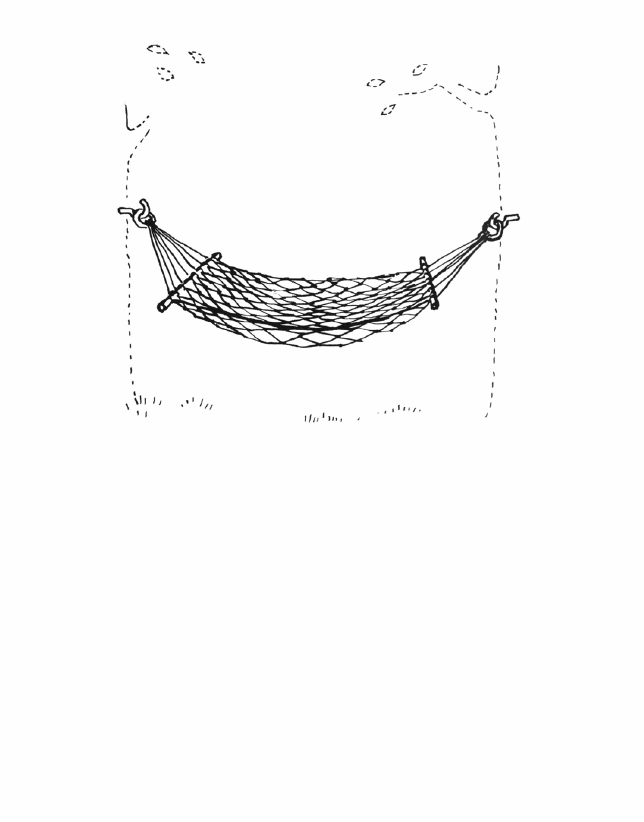


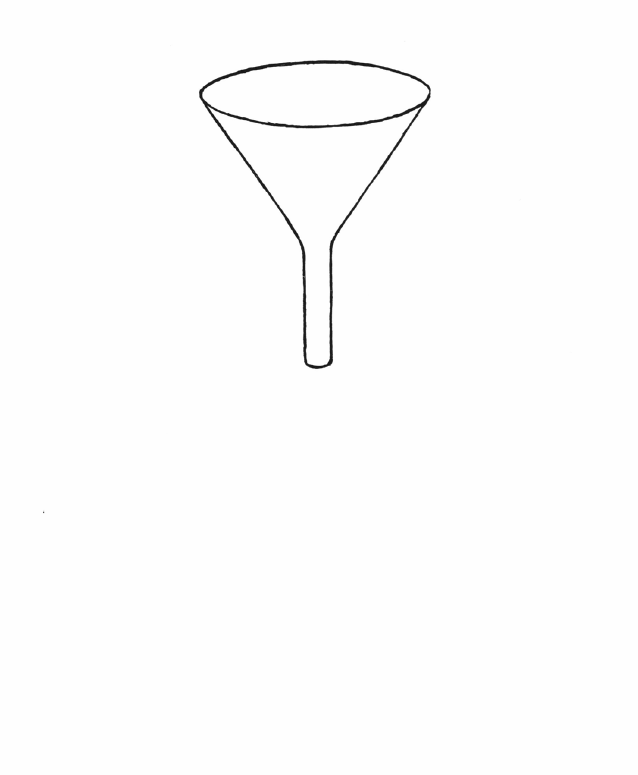


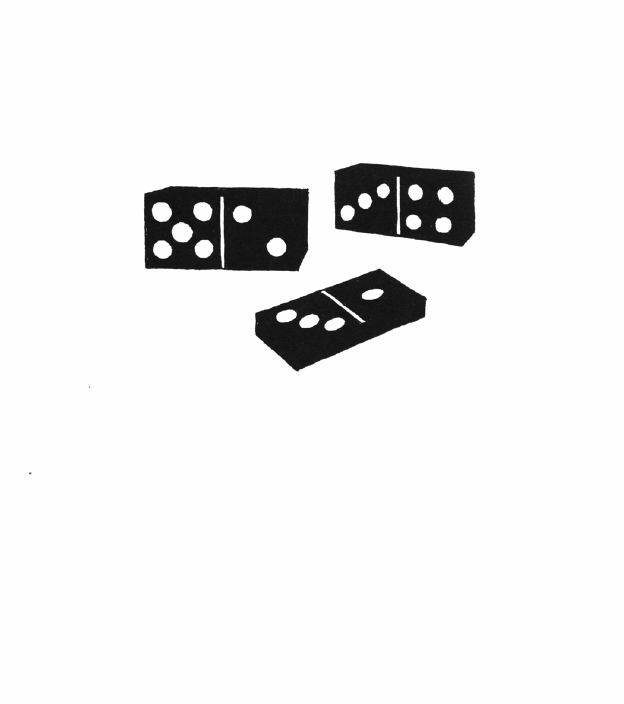


Digit Symbol Substitution Test

Brief Cognitive Battery

Figures test (Nitrini et al., 1994, 2004)

Visual Perception and Naming

Show the paper containing the 10 pictures and ask*, ‘What objects are shown in these pictures?’*

Correct perception:

Correct naming:

Incidental Memory

Hide the pictures and ask, *‘What pictures did I just show you?’ (Maximum recall time: 60 seconds)*. Document the recall order on the answer sheet.

Immediate Memory

Show the pictures again for 30 seconds, and say:

*‘Look closely and try to memorise these pictures.’* (If there is significant visual deficit, have them memorise the words you are going to say; say the names of the objects slowly, one name per second; say the whole series twice).

Hide the pictures and ask, *‘What pictures did I just show you?’ (Maximum recall time: 60 seconds).* Document the recall order on the answer sheet.

Learning

Show the pictures again for 30 seconds, and say:

*‘Look closely and try to memorize these pictures.’* (If there is significant visual deficit, have them memorise the words you are going to say; say the names of the objects slowly, one name per second; say the whole series twice).

Hide the pictures and ask, *‘What pictures did I just show you?’* (Maximum recall time: 60 seconds).
Document the recall order on the answer sheet.

*Interrupt the test, returning to delayed recall and recognition after the verbal fluency and clock drawing tests.*

Verbal Fluency Test

*‘You must say all the animal names you can remember, in the shortest time possible. You may begin.’*

Write down the number of animals recalled in 1 minute:

Clock Drawing (Sunderland et al., 1989)

Give a blank sheet of paper and say*, ‘Draw a large circle like the face of a clock and then put the clock numbers inside it’.*

*‘Set the hands at 2 hours and 45 minutes.’* (Save the drawing with the chart).

Assessment 10-6 Clock and numbers are correct.

| 10 | right time |
| --- | --- |
| 9 | slight hand disturbance (e.g. hour hand on 2) |
| 8 | more serious disturbances related to the clocks’ hands (e.g. set at 2:20) |
| 7 | completely wrong setting of the clocks’ hands |
| 6 | inappropriate use (e.g. use of digital code or circles involving numbers) |

Assessment: 5-1 Clock and numbers are incorrect.

| 5 | numbers are in reverse order or clustered in some part of the clock |
| --- | --- |
| 4 | numbers missing or outside the clock limits |
| 3 | numbers and clock no longer connected. Absence of hands |
| 2 | some evidence of having understood the instructions but with vague resemblance to a watch |
| 1 | has not attempted or succeeded in representing a clock |

Resume and complete the picture test.

Delayed recall

*‘What pictures did I show you 5 minutes ago?’* If necessary, reinforce by saying that the pictures were presented on a laminated sheet of paper. (60 seconds). Document the order of recall on the answer sheet.

##### Recognition

Show the 20 pictures and say: *‘Here are the pictures I showed you today, along with some new ones; I want you to tell me which ones you saw a few minutes ago’.* The score is calculated by subtracting the number of correct answers from the number of incorrect ones. Document the order of recall on the answer sheet.

Answer Sheet - Picture Memory Test

Incidental M. Immediate M. Learning 5-min M. Recognition

| **Shoe** |  |  |  |  |  |
| --- | --- | --- | --- | --- | --- |
| **House** |  |  |  |  |  |
| **Comb** |  |  |  |  |  |
| **Key** |  |  |  |  |  |
| **Plane** |  |  |  |  |  |
| **Bucket** |  |  |  |  |  |
| **Turtle** |  |  |  |  |  |
| **Book** |  |  |  |  |  |
| **Spoon** |  |  |  |  |  |
| **Tree** |  |  |  |  |  |

**Correct**

**Intrusions**

Note: The incidental, immediate, learning, and 5-minute memory (delayed recall) scores are equal to the number of correct answers.

For recognition, the final score is attained by subtraction: correct - intrusions.

**Recognition:**

**
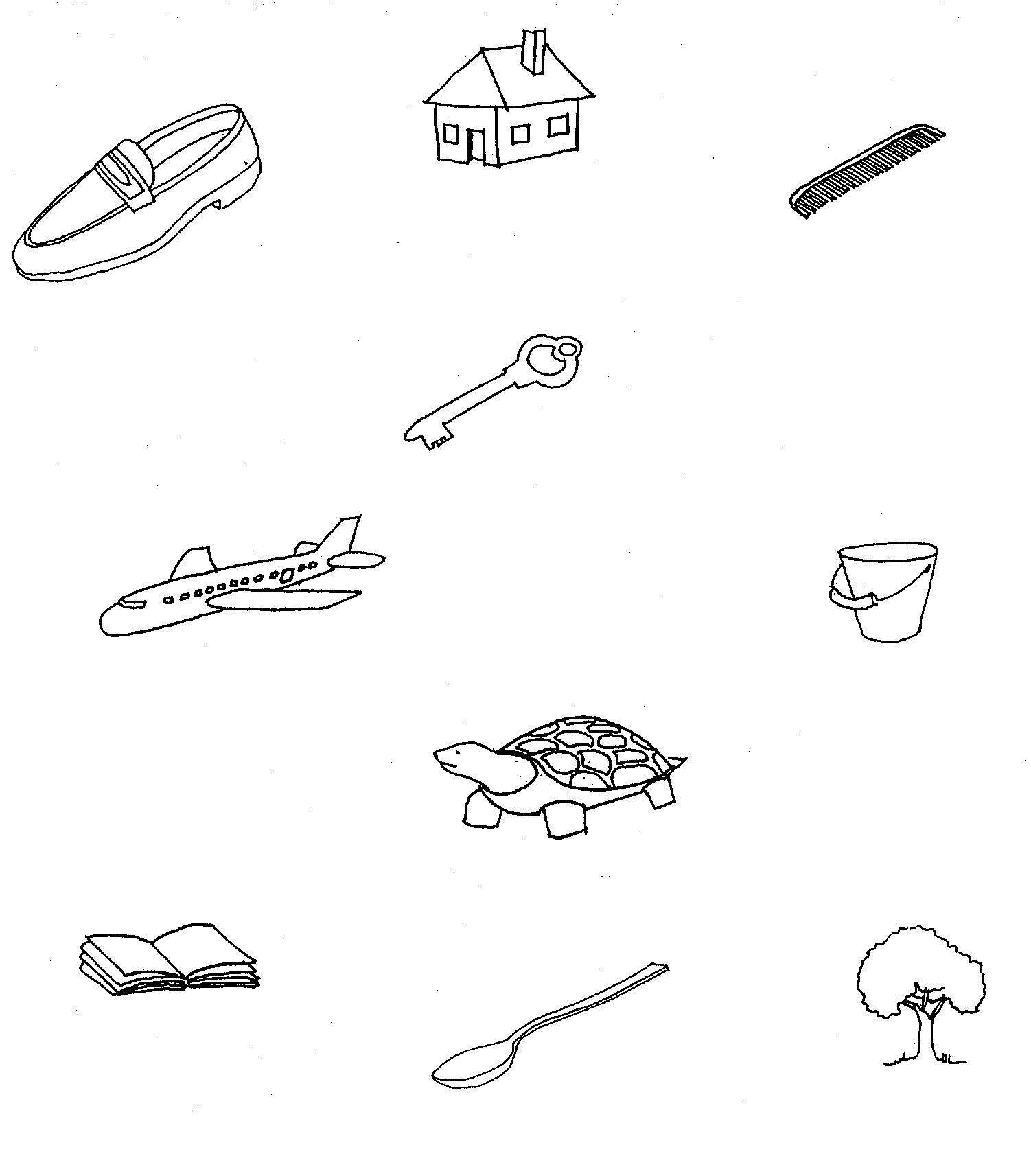
**

**
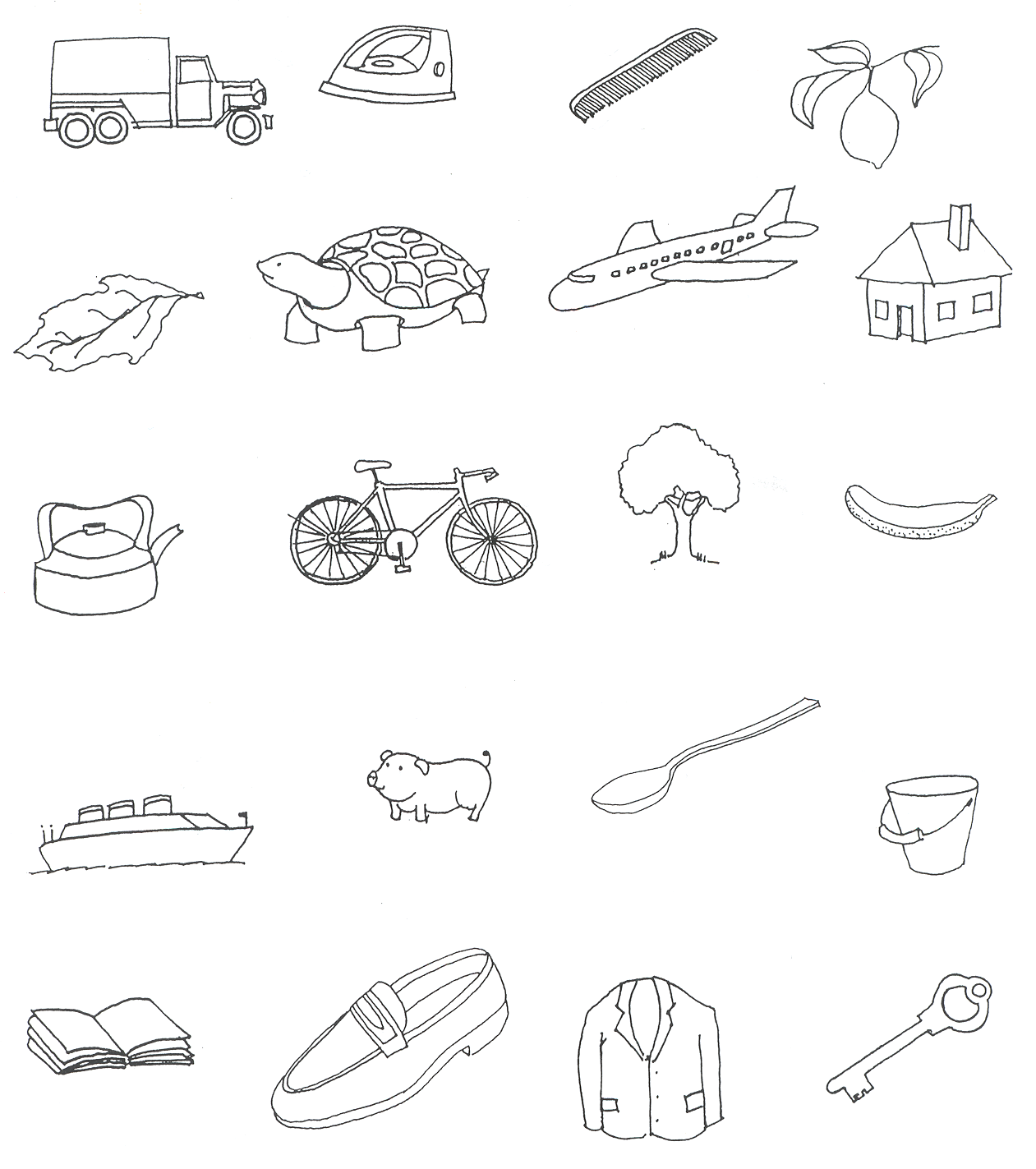
**

Behavioural Assessment: Neuropsychiatric Inventory (NPI)

The Neuropsychiatric Inventory (NPI) aims to obtain information regarding the presence of psychopathology in casualties with brain diseases. The NPI was developed for application in patients with Alzheimer's disease and other forms of dementia but may be useful in the assessment of behavioural changes in other conditions. The NPI covers 12 behavioural domains:

| 1. Delusions | 1. Apathy/Indifference |
| --- | --- |
| 1. Hallucinations | 1. Disinhibition |
| 1. Agitation/Aggression | 1. Irritation/Lability |
| 1. Depression/Dysphoria | 1. Motor disturbance |
| 1. Anxiety | 1. Night-time behaviours |
| 1. Elation/Euphoria | 1. Appetite/Eating |

The NPI is based on the responses of an informed caregiver, preferably one who lives with the patient. In the absence of an informed observer, the instrument cannot be used or should be modified. The interview is best conducted in the absence of the patient to facilitate free discussion of potentially embarrassing behaviours. Some points should be clarified when administering the NPI:

NPI Score:

Frequency is evaluated as follows:

1. Occasional - less than once a week
2. Regular - about once a week
3. Frequent - several times a week but not daily
4. Very frequent - daily or continuously present

Severity is assessed as follows:

1. Slight - produces little discomfort in the patient
2. Moderate - more upsetting for the patient but can be redirected by the caregiver
3. Severe - very upsetting for the patient and difficult to redirect

Score for each domain: Domain score = Frequency x Severity

Therefore, for each behavioural domain, there are four scores:

1. Frequency
2. Severity
3. Total (Frequency x Severity)

The total NPI score is given by the sum of all scores. Individual and total scores can be generated from the NPI.

A) DELUSIONS (NA)

*Does the patient believe things that you know are not real? For example, do they insist that someone is trying to harm or rob them? Do they claim that their relatives are not who they say they are or that the house they live in is not theirs? I am not referring only to distrust; I am interested in verifying if the patient is convinced that these things are happening to them.*

NO (go to next screening question) YES (go to sub-questions)

1. Does the patient believe they are in danger, i.e. that others are planning to hurt them?
2. Does the patient believe they are being robbed?
3. Does the patient believe that they are being cheated by their spouse?
4. Does the patient believe that unwanted guests are living in their home?
5. Does the patient believe that their spouse or other people are not who they claim to be?
6. Does the patient believe that their home is not their home?
7. Does the patient believe that their relatives plan to abandon them?
8. Does the patient believe that characters from television or magazines are present in their home? (Do they try to talk or interact with them?)

9. Does the patient believe other strange things we have not discussed?

B) HALLUCINATIONS (NA)

*Does the patient see or hear things? Do they seem to see, hear, or feel things that are not present? With this question, I am not referring only to false beliefs, such as stating that someone who has died is still alive. Rather, we want to know if they indeed experience abnormal perceptions of sounds or visions.*

NO (go to next screening question) YES (go to sub-questions)

1. Does the patient report hearing voices or act as if they hear voices?

2. Does the patient talk to people who are not there?

3. Does the patient report seeing things that others do not see or behave as though they see things that others do not (people, animals, lights, etc.)?

4. Does the patient claim to detect smells that are not perceived by others?

5. Does the patient say they feel things touching or crawling across their skin?

6. Does the patient report taste sensations without any apparent cause?

7. Does the patient describe any other unusual sensory experiences that we have not talked about?

(C) AGITATION/AGGRESSION (NA)

*Does the patient go through periods when they refuse to cooperate or will not let others help them? Are they difficult to manage?*

NO (go to next screening question) YES (go to sub-questions)

1. Does the patient become angry with those who try to care for them or resist activities such as bathing or changing clothes?

2. Is the patient stubborn, only doing what they want?

3. Is the patient uncooperative, refusing help from others?

4. Does the patient exhibit any other behaviours that make them difficult to manage?

5. Does the patient scream or swear in anger?

6. Does the patient slam doors, kick furniture, or throw objects?

7. Does the patient make mention of hurting or hitting others?

8. Does the patient exhibit any other type of aggressive or agitated behaviour?

(D) DEPRESSION/DYSPHORIA (NA)

*Does the patient seem sad or depressed? Do they say they feel sad or depressed?*

NO (go to next screening question) YES (go to sub-questions)

1. Does the patient go through periods in which they cry or whine?

2. Does the patient say or act as if they are sad or in a low mood?

3. Does the patient belittle themselves or say that they feel like a failure?

4. Does the patient consider themselves to be a bad person who is worthy of punishment?

5. Does the patient seem discouraged or say they no longer have a future?

6. Does the patient consider themselves a burden to their family, thinking that their family would have a better quality of life without them?

7. Does the patient express a desire to die or talk about killing themselves?

8. Does the patient exhibit any other signs of depression or sadness?

E) ANXIETY (NA)

*Is the patient very nervous, worried, or scared for no apparent reason? Do they seem very tense and restless? Are they afraid to be away from you?*

NO (go to next screening question) YES (go to sub-questions)

1. Does the patient say they are worried about planned events?

2. Does the patient have periods of feeling shaky, unable to relax, or feeling excessively tense?

3. Does the patient have (or complain of) shortness of breath, choking, or hiccups for no apparent reason?

4. Does the patient complain of ‘butterflies in the stomach’, palpitations, or accelerated heart rate associated with nervousness (not justified by poor health)?

5. Does the patient avoid certain places or situations that make them more nervous, such as riding in a car, meeting friends, or being in crowds?

6. Does the patient become nervous and angry when separated from you (or their caregiver)? (Do they hold on to you so as not to be separated?)

7. Does the patient exhibit any other signs of anxiety?

(F) ELATION/EUPHORIA (NA)

*Does the patient seem very cheerful or happy for no apparent reason? I am not referring to the normal joy of seeing friends, getting presents, or spending time with family members. I want to know if the patient experiences a persistently abnormal good mood or finds things funny that others do not.*

NO (go to next screening question) YES (go to sub-questions)

1. Does the patient seem to feel too well or overly happy compared to the norm?

2. Does the patient find amusing and laugh at things that others do not find funny?

3. Does the patient seem to have a puerile sense of humour, with a tendency to scoff or laugh inappropriately (such as when something unfortunate happens to someone?)

4. Does the patient tell jokes or make comments that are not funny to others but seem funny to them?

5. Does the patient play pranks, such as pinching others and playing hide and seek, just for fun?

6. Does the patient boast or proclaim to have more talents or assets than they do?

7. Does the patient exhibit any other signs of feeling unreasonably high spirited or happy?

(G) APATHY/INDIFFERENCE (NA)

*Has the patient lost interest in the world around them? Have they lost interest in doing things or do they lack motivation to initiate new activities? Has it been more difficult to engage them in conversation or daily tasks? Have they been apathetic or indifferent?*

NO (go to next screening question) YES (go to sub-questions)

1. Does the patient seem less spontaneous and active than usual?

2. Has the patient been less willing to engage in conversation than before?

3. Is the patient less caring or emotional than usual?

4. Has the patient contributed less to routine household activities?

5. Does the patient seem less interested in others’ lives and plans?

6. Has the patient lost interest in friends and relatives?

7. Is the patient less enthusiastic about their usual interests?

8. Does the patient exhibit any other signs indicating that they do not care about doing new things?

(H) DISINHIBITION (NA)

*Does the patient seem to act impulsively, without thinking? Have they done or said things that should not be done or said in public? Have they done things that are embarrassing to you or others?*

NO (go to next screening question) YES (go to sub-questions)

1. Does the patient act impulsively, without considering the consequences?

2. Does the patient talk to strangers as if they know them?

3. Does the patient say harsh things to others that may hurt them?

4. Does the patient make rude remarks and/or sexual comments they would not normally make?

5. Does the patient speak openly about very personal or private matters that they would not normally bring to the public's attention?

6. Does the patient take liberties such as touching or hugging others in a way that is out of their usual character?

7. Does the patient exhibit any other signs of loss of control over their impulses?

(I) IRRITABILITY/LABILITY (NA)

*Does the patient become irritable and upset easily? Does their mood fluctuate greatly? Are they unusually impatient? I am not referring to frustration over memory loss or inability to perform routine tasks; I want to know whether the patient has been abnormally irritable and impatient or presents sudden emotional oscillations different from their norm.*

NO (go to next screening question) YES (go to sub-questions)

1. Is the patient often in a bad mood characterised by overreacting to minor things?

2. Does the patient suddenly change moods, from polite one moment to angry the next?

3. Does the patient present unpredictable flashes of anger?

4. Is the patient intolerant, complaining of delays or tardiness regarding scheduled activities?

5. Is the patient moody and irritable?

6. Does the patient argue at random, making it difficult to deal with them?

7. Does the patient exhibit other signs of irritation?

J) MOTOR DISTURBANCE (NA)

*Does the patient wander about aimlessly doing repetitive things like opening and closing drawers or cupboards, rummaging around repeatedly, or tying knots in strings and shoelaces?*

NO (go to next screening question) YES (go to sub-questions)

1. Does the patient wander around the house for no apparent reason?

2. Does the patient go through drawers or cabinets?

3. Does the patient repeatedly dress and undress?

4. Does the patient perform repetitive activities habitually?

5. Does the patient engage in repetitive activities, such as manipulating their buttons, fiddling with things, tying knots on strings, etc.?

6. Does the patient move around a lot, i.e. cannot sit still and/or constantly fidgets by tapping their feet or fingers?

7. Does the patient perform any other activity repetitively that we have not mentioned?

L) NIGHT-TIME BEHAVIOURS s (NA)

*Has the patient had difficulty sleeping? (Do not include getting up once or twice at night to go to the bathroom and then going right back to sleep.) Do they stay up at night? Do they wander at night, dress themselves, or disturb your sleep?*

NO (go to next screening question) YES (go to sub-questions)

1. Does the patient have difficulty falling asleep? '

2. Does the patient get up at night? (Do not include getting up once or twice at night to go to the bathroom and then going right back to sleep.)

3. Is the patient wandering, pacing, or engaging in inappropriate activities at night?

4. Does the patient wake you up at night?

5. Does the patient wake up, get dressed, and mention going out, believing that morning has come and it is time to start the day?

6. Does the patient wake up too early in the morning (before their usual time)?

7. Does the patient sleep too much during the day?

8. Does the patient present any other troublesome nocturnal behaviour that we have not discussed?

M) APPETITE/EATING (NA)

*Did the patient have any disturbances in appetite, weight, or diet? (Consider this to be NA if they are incapacitated and need to be fed). Has there been any difference in their food preferences?*

NO (go to next screening question) YES (go to sub-questions)

1. Has the patient had less of an appetite?

2. Has the patient had more of an appetite?

3. Has the patient lost weight?

4. Has the patient gained weight?

5. Has the patient exhibited any changes in eating behaviour, such as putting too much food in their mouth at once?

6. Has the patient shown any change in the type of food they like, such as excessively consuming sweets or other specific types of food?

7. Has the patient developed new eating behaviours, such as eating exactly the same types of food every day or eating food in exactly the same order?

8. Has the patient presented with any other appetite or feeding alterations that we did not discuss?

GERIATRIC DEPRESSION SCALE (15 ITEMS)

1. Are you basically satisfied with your life?

2. Have you dropped many of your interests and activities?

3. Do you feel that your life is empty?

4. Do you often get bored?

5. Are you in good spirits most of the time?

6. 6. Are you afraid that something bad is going to happen to you?

7. Do you feel happy most of the time?

8. Do you often feel helpless?

9. Do you prefer to stay at home rather than go out and doing new things?

10. Do you feel you have more memory problems than most people?

11. Do you think it is wonderful to be alive now?

12. Do you feel pretty worthless the way you are now?

13. Do you feel full of energy?

14. Do you feel that your situation is hopeless?

15. Do you think that most people are better off than you are?

Note: The scale can be self-applied for individuals with more than 4 years of schooling, who have good reading comprehension skills. For individuals with up to 4 years of schooling, the examiner should always read the scale to them (without interpreting or explaining in detail). The GDS-15 should not be applied in the presence of an accompanying family member or friend. Assign 1 point for each response suggesting depression.

Interpretation:

- Score > 8 indicates a probable diagnosis of major depression.

Questionnaire on Cognitive Decline in the Elderly

IQCODE - Informant Questionnaire on Cognitive Decline in the Elderly, with 16 items (Jorm, 1994). We want you to recall what your friend or relative was like 10 years ago and compare it to what they are like now. Ten years ago was in 20__. Below are situations in which this person has to use their memory or intelligence. We want you to indicate whether these abilities have improved, stayed the same, or worsened in the described situations over the past 10 years. Note the importance of comparing their present performance with that of 10 years ago. So, if 10 years ago, this person always forgot where they had left things, and they still do, then the appropriate answer would be ‘not much change’. Please indicate the changes you have observed by marking an X in the appropriate column.


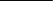


| Compared to 10 years ago, how is this person at: | Much improved | A bit improved | Not much change | A bit worse | Much worse |
| --- | --- | --- | --- | --- | --- |
| 1. Remembering things about family and friends, e.g. occupations, birthdays, addresses |  |  |  |  |  |
| 2. Remembering things that have happened recently |  |  |  |  |  |
| 3. Recalling conversations a few days later |  |  |  |  |  |
| 4. Remembering their address and telephone number |  |  |  |  |  |
| 5. Remembering what day and month it is |  |  |  |  |  |
| 6. Remember where things are usually kept |  |   |  |  |  |
| 7. Remember where to find things which have been put in a different place than usual |  |  |  |  |  |
| 8. Knowing how to work familiar machines around the house |   |  |  |  |  |
| 9. Learning to use a new gadget or machine around the house |   |  |  |  |  |
| 10. Learning new things in general |   |  |  |  |  |
| 11. Following a story in a book or on TV |   |  |  |  |  |
| 12. Making decisions related to everyday problems |     |  |  |   |  |
| 13. Handling money for shopping |  |  |  |  |  |
| 14. Handling financial tasks, e.g. pension, dealing with the bank |  |  |  |  |  |
| 15. Handling other everyday arithmetic tasks, e.g. how much food to buy, knowing the length of time between visits from family or friends |  |  |  |  |  |
| 16. Using their intelligence to understand what is going on and to reason things through |   |  |  |  |  |
|  |   |  |  |  |  |

Specification of Computerized Neuropsychological Tests - GIRAF Study

# Selective Visual Attention Test

The selective visual attention test, illustrated in Fig. 1, consists of the presentation of 60 frames of stimuli, between which there are no intervals, each containing a target stimulus at the top of the screen and a set of three stimuli 2 cm below it. The stimuli were selected from the Adobe Photoshop image library (<http://www.adobe.com/>). Variability between frames was sought, with approximately 3 cm on the vertical and horizontal axes. The individual's task is to indicate, as quickly as possible, whether the target stimulus is present in the set of stimuli by pressing 1 for ‘yes’ and 2 for ‘no’. Each stimulus frame remains on the screen for a maximum of 5 milliseconds. If there is no response from the subject within this period, the next frame is immediately presented. The average duration of the test is 2 minutes. The variables measured are reaction time and accuracy across a total of 60 trials. This test, which consists of a visual search task, aims to assess the ability to direct attention to a given stimulus.


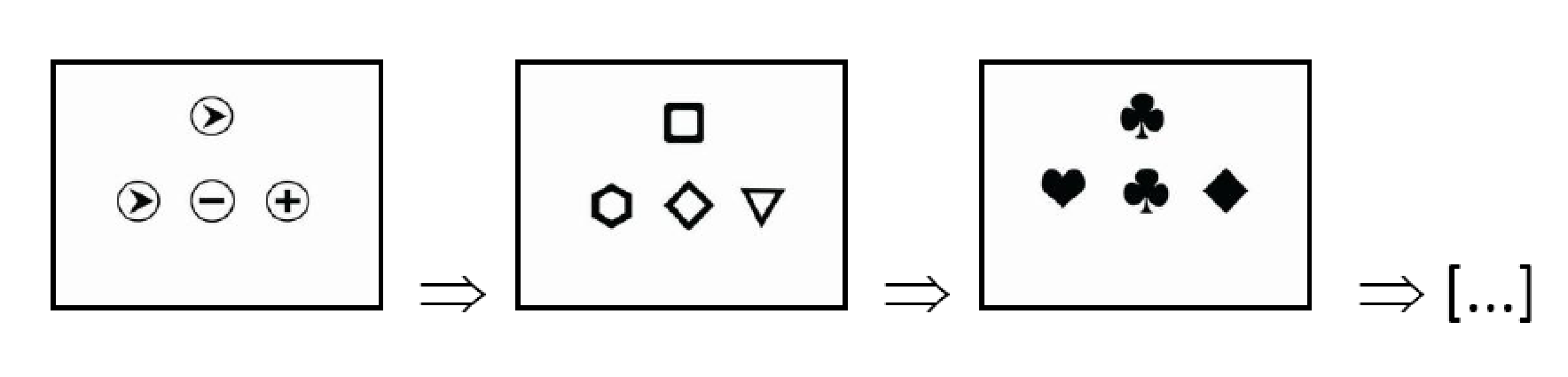


*FIGURE 1 - Illustrative scheme of the selective visual attention test.*

# Divided Visual Attention Test

The divided visual attention test, illustrated in Fig. 2, consists of the presentation of words (nouns) at the centre of the screen and a picture of a star 6 cm above the central point. The words are presented in Arial font, size 30, and the figure has 3 cm on the vertical and horizontal axes. Throughout the test, 60 frames of stimuli are displayed, with no intervals. Thirty pictures are of objects whose names begin with the letter ‘c’, and the remainder are of objects whose names begin with other letters. Each of the 60 pictures may or may not show the picture. Subjects' task is to indicate, as quickly as possible, whether or not the word begins with the letter ‘c’ by pressing 1 for ‘yes’ or 2 for ‘no’ (Task 1) and saying ‘star’ whenever the picture appears on the screen (Task 2). Throughout the test, the figure is displayed 10 times. The examiner records the responses separately. Each stimulus frame remains on the screen for a maximum of 5 milliseconds. If there is no response from the subject within this period, the next frame is immediately presented. The average duration of the test is 2 minutes. The variables measured are reaction time and accuracy in Task 1 across a total of 60 trials and accuracy in Task 2. This test, which consists of a dual task, aims to assess the ability to divide attention between two visual stimuli.


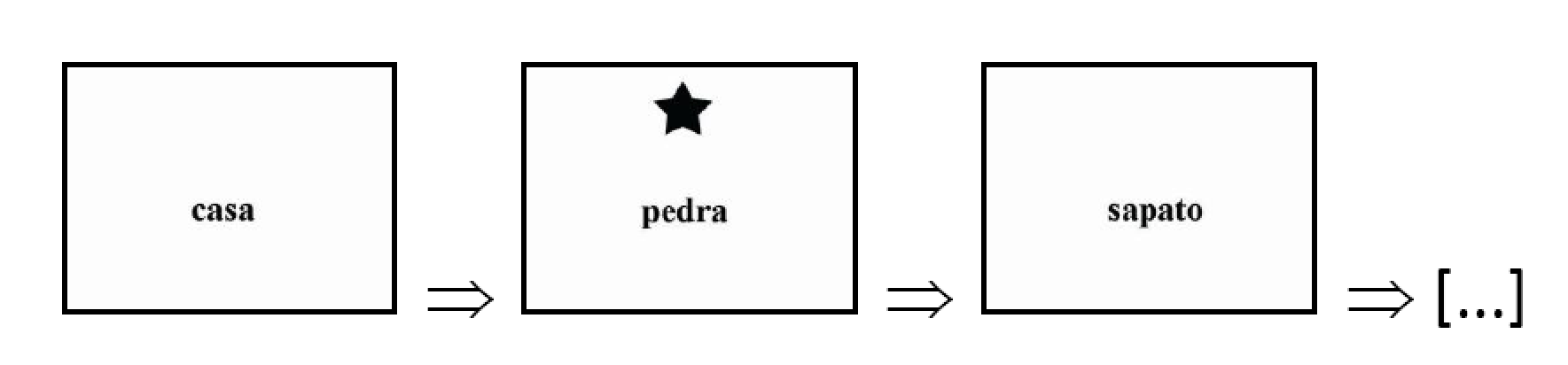


*FIGURE 2 - Illustrative scheme of the divided visual attention test.*

# Sustained Visual Attention Test

The sustained visual attention test, illustrated in Fig. 3, consists of the presentation, at the centre of the screen, of the numbers 1, 2, or 3, along with the alternating presentation of the symbols + and =, with gradually increasing frequency. The stimuli have approximately 2 cm on the vertical and horizontal axes. Each symbol is displayed 43 times during the first portion of the test, lasting 600 milliseconds; 51 times during the second portion, lasting 500 milliseconds; and 64 times during the third portion, lasting 400 milliseconds. During each of the test segments, the numbers 1, 2, or 3 are presented 18 times, for a total of 54 displays. There are no intervals between the appearance of symbols or between the appearance of symbols and numbers. The three test segments occur sequentially, with no intervals between them. The individual's task is to respond, as quickly as possible, to the appearance of the numbers by pressing the corresponding number keys. Each number remains on the screen for a maximum of 5 milliseconds. If there is no response from the subject within this period, the next stimulus (+ or =) is immediately presented. The average duration of the test is 4 minutes. The variables measured are reaction time and accuracy across a total of 54 trials. This test aims to assess the ability to maintain visual attention for a longer period of time.


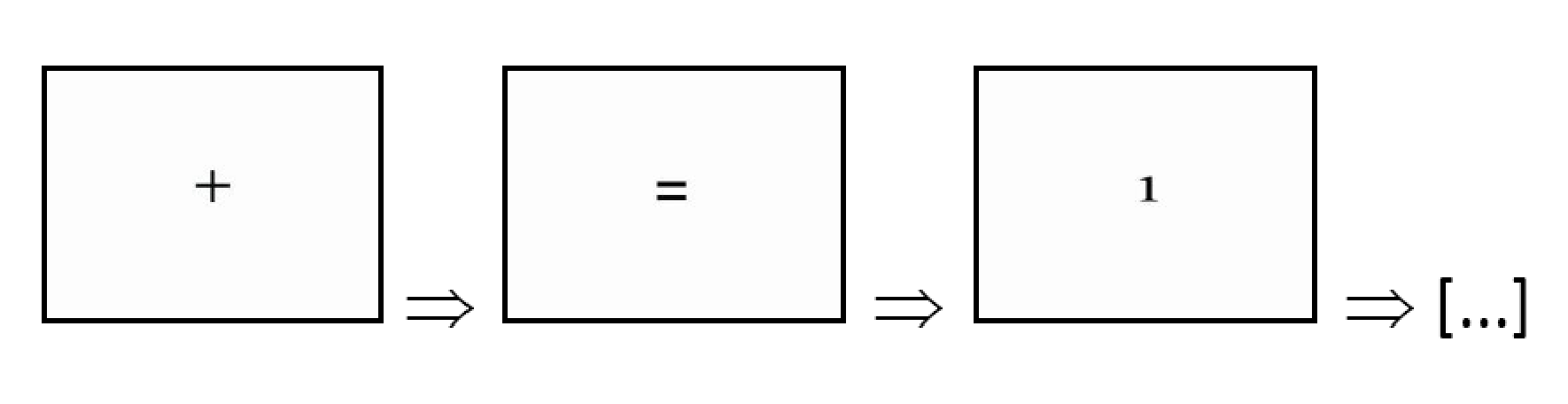


*FIGURE 3 - Illustrative scheme of the sustained visual attention test.*

# Simple Reaction Time Test

The simple reaction time test, illustrated in Fig. 4, consists of the presentation of a square at the centre of the screen at varied time intervals (between 500 and 2.5 milliseconds). This picture has 2.5 cm in the vertical and horizontal axes. Throughout the test, the picture is presented 40 times. The individual's task is to respond as quickly as possible to the appearance of the picture by pressing 1. The stimulus remains on the screen for a maximum of 3 milliseconds. If the subject does not respond within this period, the next stimulus is presented after a variable time interval. The average duration of the test is 1 minute. The variable measured is reaction time across a total of 40 trials.


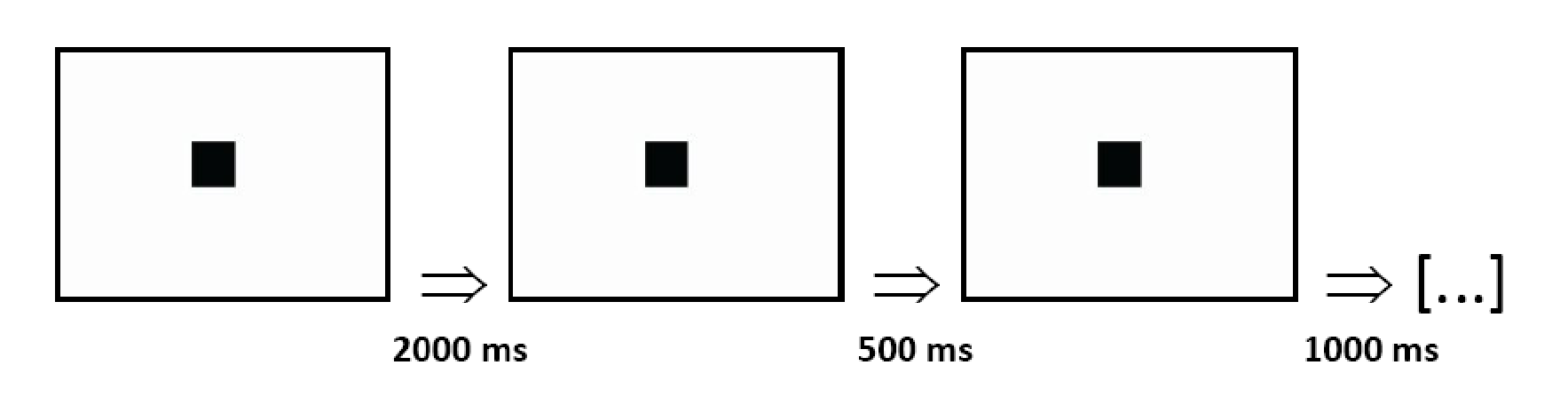


*FIGURE 4 - Illustrative scheme of the simple reaction time test.*

Each neuropsychological test described is preceded by standardised instructions that are displayed on the computer screen regarding the task to be performed. After the presentation of instructions, all tests are also preceded by a short training session to familiarise the subject with the task to be performed.

**Table S3**. Mean change from baseline according to TTR subgroups (< 70% and ≥ 70%) in the warfarin group.

| Cognitive  Assessment | TTR < 70  Mean  (SD) N | | TTR ≥ 70  Mean  (SD) N | | Contrast (95% CI) | P-value | Cohen's d Effect Size |
| --- | --- | --- | --- | --- | --- | --- | --- |
| MMSE | -0.59  (-1.30 to 0.12) | 33 | -0.77  (-1.49 to -0.06) | 33 | -0.19 (-1.20 to 0.83) | 0.72 | -0.09 |
| MoCA | 0.54  (-0.35 to 1.43) | 33 | 0.67  (-0.22 to 1.56) | 33 | 0.14  (-1.13 to 1.41) | 0.83 | 0.05 |
| NTB | -0.04  (-0.17 to 0.08) | 33 | 0.04  (-0.09 to 0.17) | 33 | 0.08  (-0.10 to 0.26) | 0.37 | 0.22 |
| CGNT score |  |  |  |  |  |  |  |
| Composite Score | -0.009  (-0.18 to 0.16) | 26 | -0.006  (-0.16 to 0.15) | 29 | 0.003  (-0.23 to 0.24) | 0.98 | 0.006 |
| Simple Reaction Time (AC) | 0.19  (-0.19 to 0.58) | 29 | 0.05  (-0.34 to 0.43) | 30 | -0.15  (-0.70 to 0.40) | 0.59 | -0.14 |
| Simple Reaction Time (RT) | -188  (-256 to -119) | 29 | -171  (-239 to -104) | 30 | 16.1  (-81 to 113) | 0.74 | 0.08 |
| Sustained Attention (AC) | -1.57  (-3.38 to 0.26) | 28 | -0.69  (-2.48 to 1.09) | 29 | 0.872  (-1.78 to 3.52) | 0.51 | 0.19 |
| Sustained Attention (RT) | -45.04  (-132.60 to 42.50) | 28 | 7.14  (-78.80 to 93.10) | 29 | 52.2  (-72.20 to 177) | 0.40 | 0.23 |
| Selective Attention  (AC) | 1.51  (-5.82 to 8.85) | 28 | 4.17  (-2.91 to 11.26) | 30 | 2.66  (-7.62 to 12.90) | 0.61 | 0.14 |
| Selective Attention (RT) | -194  (-522 to 133.10) | 28 | -260  (-576 to 56.30) | 30 | -65.50  (-525 to 394) | 0.78 | -0.08 |
| Divided Attention (AC) | -0.07  (-2.26 to 2.12) | 28 | 0.54  (-1.57 to 2.66) | 30 | 0.62  (-2.49 to 3.72) | 0.69 | 0.11 |
| Divided Attention (RT) | -16.90  (-126 to 91.70) | 28 | -12.60  (-117 to 92.20) | 30 | 4.32  (-149 to 158) | 0.95 | 0.01 |

MMSE: Mini-Mental State Exam; MoCA: Montreal Cognitive Assessment; NTB: composite neuropsychological test battery consisting of the average of the z-scores for Boston naming test, semantics verbal fluency, phonemic verbal fluency, trail making tests, clock-drawing test, and digit symbol substitution test; CGNT: a composite score composed of the average of the z-scores of the following computer-generated neuropsychological tests: Simple Reaction Time, Sustained Attention, Selective Attention, and Divided Attention; AC: accuracy; RT: reaction time

**Additional statistical analysis**

**Multiple imputation**

To assess the potential impact of missing data in our analyses we visually inspected distributions of relevant variables according to missingness of post-treatment score values and then performed a regression-based multiple imputation analysis using Predictive Mean Matching as implemented in the MICE R package.

The table below shows the missing value percentages for each score regarding baseline and post-treatment test. Only CGNT scores have missing baseline values due to incorrect application of the test in a few random patients at the initial of trial that resulted in discarded results.

**Table S4.** Missing value percentages for each score regarding baseline and post-treatment test

| Score | Missing Count (Percentage) | |
| --- | --- | --- |
|  | Baseline | Post-Treatment |
| MMSE | 0 (0%) | 34 (18.58%) |
| MoCA | 0 (0%) | 34 (18.58%) |
| NTB | 0 (0%) | 34 (18.58%) |
| CGNT | 24 (13.11%) | 44 (24.04%) |

The table below shows the patterns of cooccurring missing values for some the score variables. Yes/No denote missing/not-missing value of the respective score. We observe that as expected the missingness of outcome variables is highly associated, as they are caused by loss of follow-up evaluations. The other meaningful pattern is that of patients with missing baseline value of CGNT but observed post-treatment value. As commented, this was caused by the deletion of data from some patients due to misapplication of the baseline evaluation.

**Table S5**. Missing value percentages for each score regarding baseline and post-treatment test

| Count | MMSE  Post | MoCA Post | NTB Post | CGNT Baseline | CGNT Post |
| --- | --- | --- | --- | --- | --- |
| 123 | No | No | No | No | No |
| 7 | No | No | No | No | Yes |
| 1 | Yes | Yes | Yes | No | No |
| 28 | Yes | Yes | Yes | No | Yes |
| 15 | No | No | No | Yes | No |
| 4 | No | No | No | Yes | Yes |
| 5 | Yes | Yes | Yes | Yes | Yes |

We also analyzed if there is noticeable bias in the distributions of covariate variables used in the linear regression analysis for the groups with missing and non-missing values. The figure below shows the distributions of Age, log Education and Baseline scores for the groups with observed and missing values of the post-treatment MMSE score. The red curve show a fitted gaussian whose mean and standard deviation annotate the plots. Overall, we see no pronounced difference between distribution of values in the observed/missing data portions. The only noticeable deviation is the slightest increase in mean age for the patients with missing follow-up evaluation.

**Figure S4.** Distributions of Age, log Education and Baseline scores for the groups with observed and missing values of the post-treatment MMSE score.


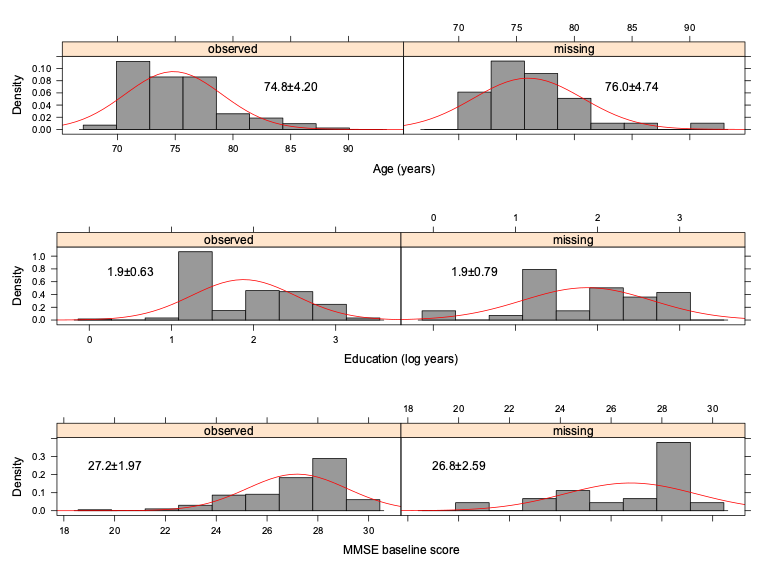


The figures below show the distributions of Age, log Education and Baseline scores for MoCA. Once more, we see no pronounced difference in the segments, with the only noticeable difference for the age distribution.

**Figure S5.** Distributions of Age, log Education and Baseline scores for the groups with observed and missing values of the post-treatment MMSE score.


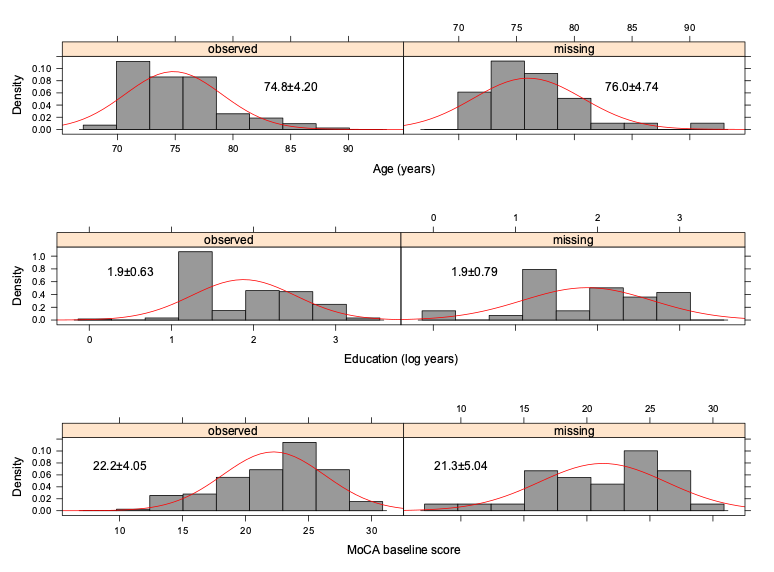


The plots below refer to the NTB score. While the differences in distributions with respect to missingness are again not very pronounced, we see here a most relevant difference in the distribution of baseline score values, which exhibit a higher left skewedness (i.e., a skew towards negative values for missing values of post-treatment NTB score) that is less pronounced in the segment of observed post-treatment scores. These graphs suggest that we cannot discard the possibility of a link between drop-out and baseline NTB score.

**Figure S6.** The plots below refer to the NTB score.


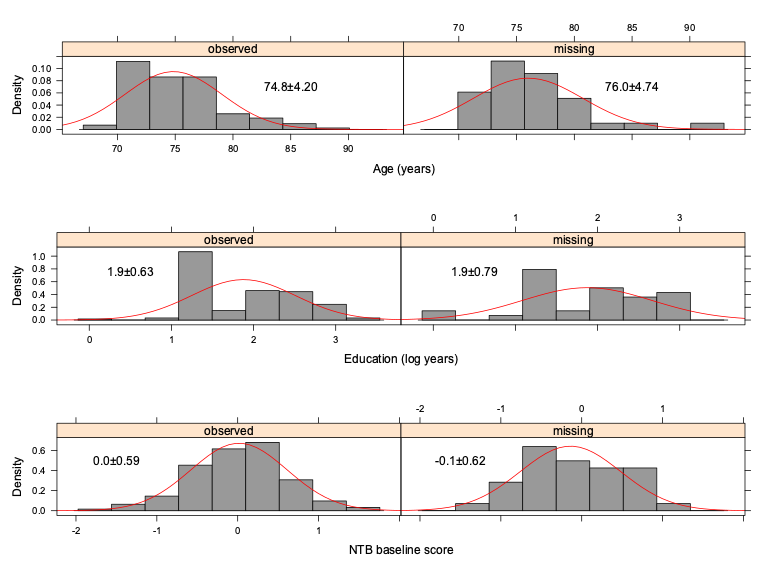


The plots below show distribution of Age and Log Education segmented by missingness of baseline CGNT scores. We observe no pronounced difference.

**Figure S7**. The plots below show distribution of Age and Log Education segmented by missingness of baseline CGNT scores.


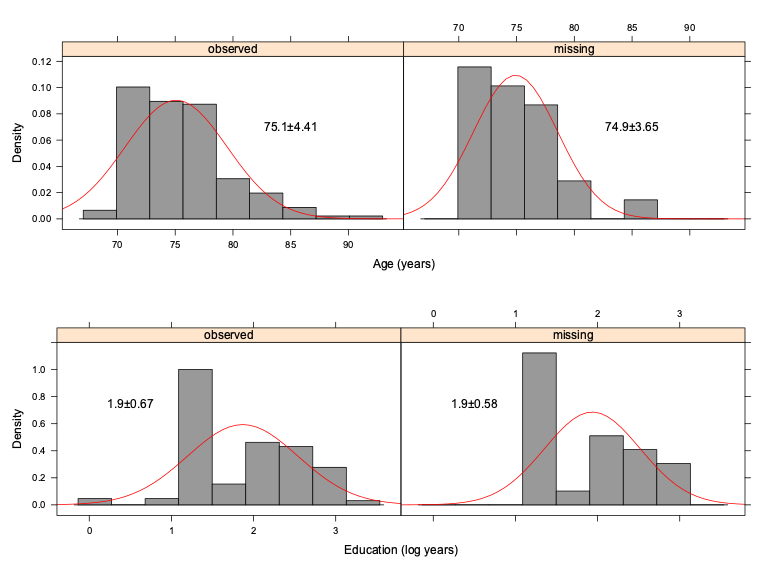


Last, we show histograms for Age, Log Education and Baseline score values according to missingness of post-treatment values. Again, we observe no pronounced difference in the histogram pairs except for the outcome variable.

**Figure S8.** Histograms for Age, Log Education and Baseline score values according to missingness of post-treatment values


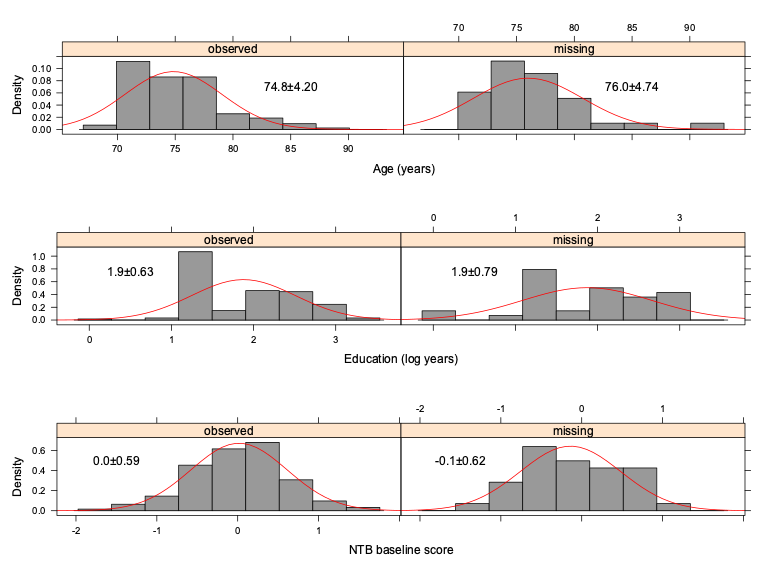


To mitigate any bias introduced by missingness of post-treatment scores (drop out patients) under a missing at random hypothesis, we carried out a regression-based multiple imputation analysis for the least-square mean values. In the following, we used 10 imputations using 30 iterations of predictive mean matching using baseline and post-treatment score values as well as Age, log Education, Sex and Group information as predictors. Convergence (mixing) of the chains were verified by visual inspection of the intertangling traces. We also observed that imputed values are distributed similarly to observed values.

The table below shows pooled descriptive statistics (Median [IQR]) for the post-treatment score values in the original and first three imputed data sets. The remaining imputed data sets show similar comparative results. We see from the table that in the imputed data sets the between-group difference of post-treatment scores is even less statistically significant (according to a Kruskall-Wallis test at 5% significance level). Indeed, in all 10 imputed datasets, no difference was deemed statistically significant.

**Table S6.** Pooled descriptive statistics (Median [IQR]) for the post-treatment score values in the original and first three imputed data sets.

| Variable | All  N=183 | Dabigatran N=96 | Warfarin N=87 | p-value | N (count) |
| --- | --- | --- | --- | --- | --- |
| MMSE Original Imputation 1 Imputation 2  Imputation 3 | 27 [25;29]  27 [25;29] 27 [25;29]  27 [25;29] | 27 [25;29] 27 [25;29]  27 [25;29]  27 [25;29] | 27 [25; 28.8] 27 [25;28]  27 [25;29]  27 [25;29] | 0.81 0.545  0.755  0.603 | 149 183  183  183 |
| MoCA Original Imputation 1  Imputation 2  Imputation 3 | 22 [20;25] 22 [19.5;25]  22 [20;25]  22 [19;25] | 23 [20;25]  22.5 [20;25]  22 [20;25]  22.5 [20;25] | 22 [19;25]  22 [19;25.5]  22 [19;26]  22 [18.5;26] | 0.536  0.793  0.997  0.684 | 149  183  183  183 |
| NTB Original Imputation 1  Imputation 2  Imputation 3 | 0.09 [-0.33;0.50]  0.07 [-0.36;0.46]  0.09 [-0.35;0.50]  0.07 [-0.35;0.55] | 0.15 [-0.21;0.55]  0.09 [-0.28;0.51]  0.09 [-0.27;0.55]  0.11 [-0.25;0.55] | -0.08 [-0.52;0.44]  -0.08 [-0.52;0.38]  0.01 [-0.52;0.46]  0.01 [-0.52;0.46] | **0.049**  0.065  0.132  0.079 | 149  183  183  183 |
| CGNT Original Imputation 1  Imputation 2  Imputation 3 | 0.24 [-0.11;0.43]  0.21 [-0.29;0.42]  0.23 [-0.26;0.41]  0.24 [-0.23;0.44] | 0.25 [-0.03;0.47]  0.24 [-0.19;0.45]  0.24 [-0.27;0.45]  0.24 [-0.12;0.45] | 0.18 [-0.27;0.39]  0.10 [-0.35;0.40]  0.21 [-0.26;0.40]  0.23 [-0.27;0.41] | **0.05**  0.192  0.467  0.377 | 139  183  183  183 |

The tables below show pooled results (using Rubin’s rule) for the linear regression analyses over the 10 imputed datasets for each score. Except for the MoCA score, we see that the Group effect is *not* statistically significantly different than 0. We also see that coefficients and p-values are similar to those of the complete-data analysis.

**Tables S7-10.** Pooled results (using Rubin’s rule) for the linear regression analyses over the 10 imputed datasets for each score

For MMSE:

| Variable | Estimate | Standard Error | p-value |
| --- | --- | --- | --- |
| Intercept | 6.841 | 4.06011 | 0.09 |
| Group:Warfarin | 0.1898 | 0.392 | 0.631 |
| MMSE (baseline) | -0.2356 | 0.1175 | 0.052 |
| Log Education | 0.993 | 0.321 | **< 0.005** |
| Age | -0.04 | 0.0461 | 0.38 |

For MoCA:

| Variable | Estimate | Standard Error | p-value |
| --- | --- | --- | --- |
| Intercept | 10.578 | 4.3558 | **0.019** |
| Group:Warfarin | 0.9292 | 0.3867 | **0.017** |
| MoCA (baseline) | -0.3132 | 0.0612 | **< 0.00001** |
| Log Education | 1.71899 | 0.378 | **< 0.0001** |
| Age | -0.0965 | 0.052 | 0.00686 |

For NTB:

| Variable | Estimate | Standard Error | p-value |
| --- | --- | --- | --- |
| Intercept | 0.27052 | 0.5771 | 0.641 |
| Group:Warfarin | -0.047237 | 0.0599 | 0.432 |
| NTB (baseline) | -0.19296 | 0.05667 | **< 0.0001** |
| Log Education | 0.175293 | 0.05479 | **< 0.005** |
| Age | -0.00741 | 0.00775 | 0.343 |

For CGNT:

| Variable | Estimate | Standard Error | p-value |
| --- | --- | --- | --- |
| Intercept | 1.385 | 0.5402 | **0.0199** |
| Group:Warfarin | 0.055 | 0.0654 | 0.403 |
| CGNT (baseline) | -0.4565 | 0.0608 | **< 0.0001** |
| Log Education | 0.1225 | 0.06174 | 0.0559 |
| Age | -0.02 | 0.00709 | **< 0.005** |

Finally, we obtain pooled least-square means and contrast estimators from them multiply imputed datasets. The results appear in the table below. The values are similar to those of the complete-data analysis.

**Table S11.** Pooled results (using Rubin’s rule) for the linear regression analyses over the 10 imputed datasets for each score

| Score | Dabigatran | Warfarin | Contrast |
| --- | --- | --- | --- |
| MMSE | -0.7 (0.28) | -0.51 (0.26) | -0.189 |
| MoCA | -0.34 (0.26) | 0.58 (0.28) | -0.929 |
| NTB | 0.04 (0.05) | -0.001 (0.04) | 0.047 |
| CGNT | -0.02 (0.05) | 0.07 (0.05) | -0.055 |

The figure below shows estimate and 95% confidence intervals for the contrasts (W-D) for each score. The results show no difference in conclusion: MoCA score is the only to show a (statistically significant and practical) difference of between-group change from baseline score values.

**Figure S9.** Estimate and 95% confidence intervals for the contrasts (W-D) for each score.


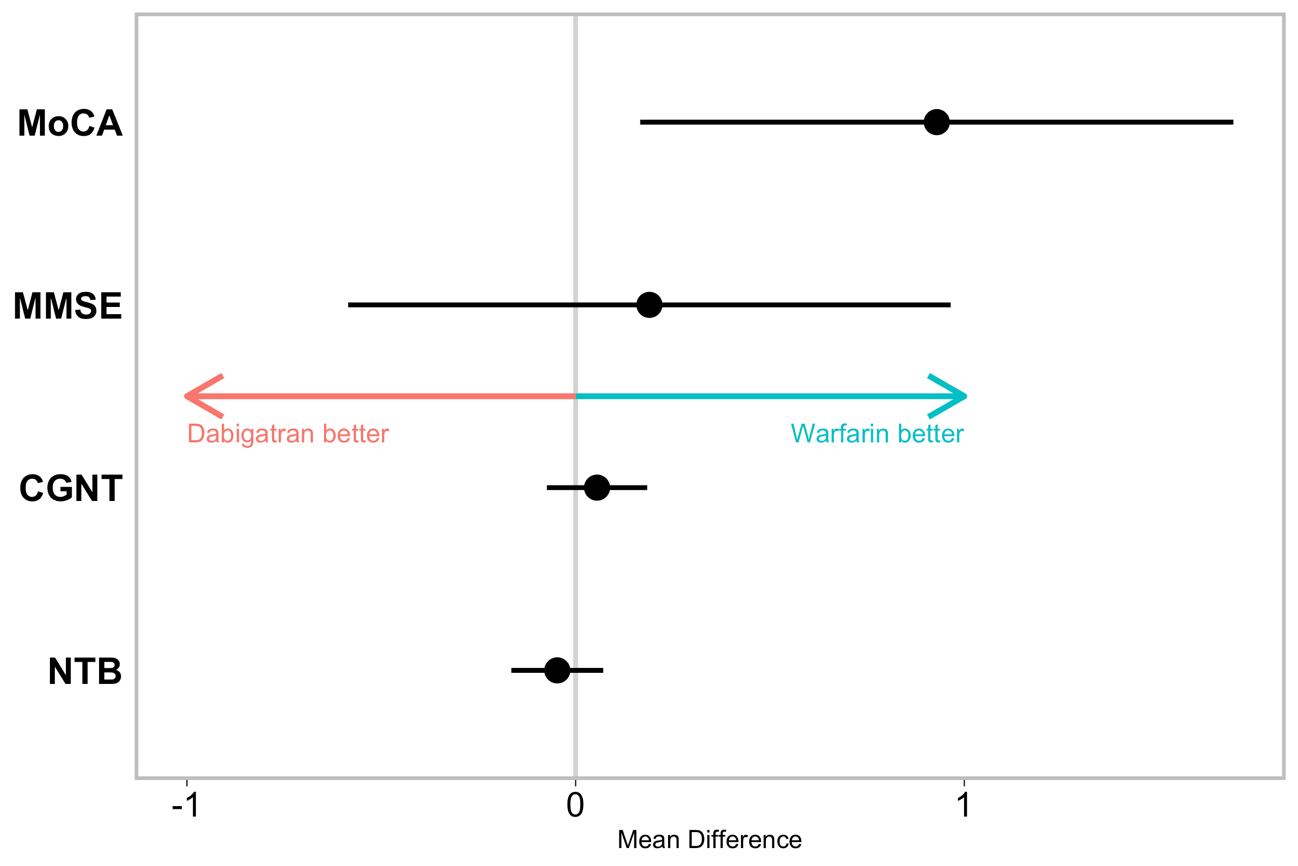


Some authors (Sullivat et al., 2018) recommend that multiple imputation be performed separately for each group, to avoid bias introduced by interaction between the group/treatment variable and the covariates (not accounted for in the analysis). This has the drawback of reducing efficiency (by increasing standard errors of the imputed analysis). To contrast either approach, we repeated the analysis while performing imputation separately in each group. We used M=30 imputations (to account for the added variance). The results are summarized in the figure below. One can see that the results are essentially identical.

**Figure S10.** Repeated analysis while performing imputation separately in each group.

**Multiple Comparison Adjustment**

To account for the reduced significance incurred by multiple hypothesis testing for the multiple endpoints, we computed adjusted p-values for the regression analyses of the group effect by using Holm’s (1979) and Hommel’s (1988) formulas, as provided in the R stats package. Empirical evidence has shown that such methods are valid when endpoints are mildly correlated (rho < 0.5), which is our case here (Pocock et al. 1987). We also performed an adjustment using the score decomposition method of Pipper, Ritz and Bisgaard, as available in the multcomp R package, that accounts for correlation among response variable in multiple linear regression analyses. These methods control the familywise error rate with different reductions in power. The results are shown in the table below. Note that the adjusted p-values all render all differences statistically insignificant (i.e., they fail to reject the null hypothesis) at 5% level. Overall, we see also a high level of agreement in the adjusted values across the different procedures.

**Table S12.** Adjusted p-values for the regression analyses of the group effect by using Holm’s (1979) and Hommel’s (1988) formulas, as provided in the R stats package.

| Outcome | Unadjusted | Holm’s adjustment | Hommel’s adjustment | Score decomposition |
| --- | --- | --- | --- | --- |
| MMSE | 0.74 | 0.9 | 0.74 | 0.99 |
| MoCA | 0.02 | 0.08 | 0.08 | 0.08 |
| NTB | 0.45 | 0.9 | 0.74 | 0.90 |
| CGNT | 0.22 | 0.66 | 0.66 | 0.61 |
